# C26-Ceramide as highly sensitive biomarker for the diagnosis of Farber Disease

**DOI:** 10.1038/s41598-017-06604-2

**Published:** 2017-07-21

**Authors:** Claudia Cozma, Marius-Ionuț Iurașcu, Sabrina Eichler, Marina Hovakimyan, Oliver Brandau, Susanne Zielke, Tobias Böttcher, Anne-Katrin Giese, Jan Lukas, Arndt Rolfs

**Affiliations:** 1 Centogene AG, Schillingallee 68, 18057 Rostock, Germany; 2 Albrecht-Kossel-Institute for Neurodegeneration, Rostock University Medical Centre, Gehlsheimerstraße 20, 18147 Rostock, Germany

## Abstract

Farber disease (FD) is a rare autosomal recessive disease caused by mutations in the acid ceramidase gene (*ASAH1*). Low ceramidase activity results in the accumulation of fatty substances, mainly ceramides. Hallmark symptoms at clinical level are periarticular nodules, lipogranulomas, swollen and painful joints and a hoarse voice. FD phenotypes are heterogeneous varying from mild to very severe cases, with the patients not surviving past their first year of life. The diagnostic aspects of FD are poorly developed due to the rarity of the disease. In the present study, the screening for ceramides and related molecules was performed in Farber affected patients (n = 10), carriers (n = 11) and control individuals (n = 192). This study has the highest number of enrolled Farber patients and carriers reported to present. Liquid chromatography multiple reaction mass spectrometry (LC/MRM-MS) studies revealed that the ceramide C26:0 and especially its isoform 1 is a highly sensitive and specific biomarker for FD (p < 0.0001). The new biomarker can be determined directly in the dried blood spot extracts with low sample consumption. This allows for easy sample preparation, high reproducibility and use in high throughput screenings.

## Introduction

Farber disease (FD) (OMIM 228000), also known as Farber’s lipogranulomatosis, is a rare autosomal recessive disease caused by mutations in the N-acylsphingosine amidohydrolase (*ASAH1*) gene (8p22). This gene encodes acid ceramidase, a lysosomal enzyme that catalyzes the terminal step in glycosphingolipid metabolism, where ceramide is degraded to sphingosine and fatty acid. Impaired acid ceramidase function leads to accumulation of ceramides in the cells and tissues, particularly around the joints^1–5^. Over 25 mutations in the *ASAH1* gene causing FD have been described, although genotype-phenotype correlations are not always obvious^6^. Recent research has revealed that deficiency of the same enzyme is responsible for a rare form of spinal muscular atrophy associated with myoclonic epilepsy (SMAPME).

At clinical level, FD manifests through a unique triad of clinical symptoms: (1) painful and progressively deformation of joints, (2) subcutaneous nodules (lipogranulomata), particularly near the joints and over pressure points, and (3) a hoarse voice or a weak cry due to laryngeal involvement. In addition, hepatosplenomegaly, rapid neurological deterioration or developmental delay are reported^1–5^. Seven clinical subtypes of the disease are described, classified according to the age of onset, symptom severity, and target tissue.

Ceramide accumulation in different organs is the defining feature of all FD subtypes^1, 2, 7–10^, but has also been implicated in the pathobiology of other diseases such as insulin resistance connected with obesity, alcohol abuse, and cancer^3, 11–13^.

Although ceramides are minor components of membranes in general, they play a key role in the mediation of various cellular events^14^. As an important second messenger, ceramides are involved in apoptotic signaling via direct interaction with and activation of different intracellular protein kinases and phosphatases^15^. Furthermore, ceramides regulate specific cell responses to stress agents and are implicated in cell differentiation, senescence and growth^16^.

The histopathological examination of postmortem tissue of Farber patients revealed granulomatous lesions, composed of spindle or oval-shaped storage cells in many organs, including liver, kidney, skin, subcutaneous tissue, periarticular regions, central and peripheral and nervous system^17, 18^. Interestingly, the accumulating ceramides are found not only in late endosomes and lysosomes, but also in mitochondria and the plasma membrane^9^.

Since the initial discovery of FD in 1957^19^, less than 100 cases have been described in the medical literature. It is suggested, however, that the actual number of cases is being underestimated due to misdiagnosis^20^. Due to the heterogeneity of FD presentation, a high percentage of cases with mild symptoms are initially diagnosed as juvenile idiopathic arthritis (JIA)^21, 22^. The accurate diagnosis of FD is of crucial importance, as the development of enzyme replacement therapy was announced in July 2016 (Enzyvant Sciences Ltd).

The diagnosis of FD is confirmed by histopathological examination of biopsy or autopsy tissues and/or by measuring the activity of acid ceramidase in leucocytes or cultured fibroblasts.

Targeted mass spectrometry, especially high resolution triple quad mass spectrometry has allowed detection and quantification of an increased number of ceramides or ceramide related species in different mammal samples, such as skin fibroblasts homogenates, immortalized cells homogenates, plasma, muscles and liver tissue^23–27^.

Detailed analysis of ceramides and their derivatives in dried blood spots (DBS) of affected patients and healthy controls provided us with mechanistic insights into the biochemistry of the disease and led to the discovery of a biomarker candidate for FD. This biomarker, C26:0 ceramide, has the potential to become an accurate tool for detection for FD patients and routine diagnostic.

## Results

### Screening of ceramides and ceramides derivatives using liquid chromatography-multiple reaction monitoring mass spectrometry (LC/MRM-MS)

The groups included in the screening were: normal controls (NC, n = 83), Farber patients (FP, n = 10), Farber carriers (FC, n = 11) and patients with juvenile idiopathic arthritis (JIA, n = 2). The disease causing mutations detected in the *ASAH1* gene of the investigated patients are listed in Table 1.

**Table 1:** *ASAH1* mutations found in FP and FC involved in present study (results obtained with *ASAH1* transcript NM_004315.4).

Pat-ID.	Phenotype/Diagnosis	Age	Coding effect	cDNA change	Mutation type	Locus	Allele zygosity	Reference
1	FP	juvenile	p.Trp185Arg	c.553T > C	Missense	Exon 8	Heterozygous	51
			p.Lys382Gln	c.1144A > C	Missense	Exon 13	Heterozygous	Novel*
2	FP	newborn	p.Arg349Cys	c.1045C > T	Missense	Exon 12	Homozygous	Novel*
3	FP	juvenile	p.Trp185Arg	c.553T > C	Missense	Exon 8	Homozygous	51
4	FP	juvenile	—	c.752-2A > G	Splicing	Intron 9	Homozygous	Novel*
5	FP	juvenile	p.Cys47Phe	c.140G > T	Missense	Exon 2	Homozygous	Novel*
6	FP	newborn	p.Tyr458Cys	c.458A > G	Missense	Exon 6	Homozygous	Novel*
7	FP	juvenile	p.Tyr52Cys	c.155A > G	Missense	Exon 2	Homozygous	52
8	FP	juvenile	p.Tyr52Cys	c.155A > G	Missense	Exon 2	Heterozygous	52
			p.Arg349Gly	c.1045C > G	Missense	Exon 12	Heterozygous	51
9	SMAPME	adult	—	c.173 + 1G > A	Splicing	Intron 2	Heterozygous	Novel*
			p.Lys168Asn	c.504A > C	Missense	Exon 6	Heterozygous	51
10	SMAPME	adult	p.Thr58Ala	c.172A > G	Missense	Exon 2	Heterozygous	53
			p.Thr195Ile	c.584C > T	Missense	Exon 8	Heterozygous	54
11	FC	adult	p.Pro142Thr	c.424C > A	Missense	Exon 5	Heterozygous	Novel*
12	FC	adult	p.Lys382Gln	c.1144A > C	Missense	Exon 13	Heterozygous	Novel*
13	FC	adult	p.Tyr52Cys	c.155A > G	Missense	Exon 2	Heterozygous	52
14	FC	juvenile	p.Pro142Thr	c.424C > A	Missense	Exon 5	Heterozygous	Novel*
15	FC	adult	p.Pro294Leu	c.881C > T	Missense	Exon 11	Heterozygous	Novel*
16	FC	adult	p.Pro294Leu	c.881C > T	Missense	Exon 11	Heterozygous	Novel*
17	FC	adult	p.Trp185Arg	c.553T > C	Missense	Exon 8	Heterozygous	51
18	FC	newborn	—	c.1089 + 1G > C	Splicing	Intron 12	Heterozygous	Novel*
19	FC	adult	p.Trp185Arg	c.553T > C	Missense	Exon 8	Heterozygous	51
20	FC	adult	p.Tyr52Cys	c.155A > G	Missense	Exon 2	Heterozygous	52
21	FC	juvenile	p.Trp185Arg	c.553T > C	Missense	Exon 8	Heterozygous	51

FP = Farber patients, SMAPME = spinal muscular atrophy associated with progressive myoclonic epilepsy, FC-asymptomatic Farber carriers, Newborn = patients or carriers with age up to 6 months, Juvenile = patients or carriers with age 6 months to 4 years, Adults = patients or carriers with plus 17 years old. *CentoMD (https://www.centomd.com).

In a preliminary step we used LC/MRM-MS to screen for different ceramides and their derivatives (dihydroceramides, glucosylceramides, sphingomyelins, and lactosylceramides) expecting to identify biomarkers for FD. For the quantification of different targeted ceramide compounds, pure standards were purchased as follows: ceramides (C6:0, C12:0, C14:0, C16:0, C18:0, C24:0, C24:1:0, C26:0), dihydroceramides (C12:0, C18:0; C24:0), glucosylceramides (C16:0, C18:0), sphingomyelins (C12:0, C18:0), lactosylceramides (C12:0). Lyso-lactosylceramide (lyso-Gb2), ceramide C25:0 and ceramide C17:0 were used as internal standards. All the standards were characterized by high resolution mass spectrometry and LC/MRM-MS. The presence of the targeted ceramides in DBS extracts was confirmed by high resolution mass spectrometry (MS and MS^e^). The detailed method description and conditions are added in the supplementary information (Supplemental Tables S1–S8). The results of the ceramide screening are summarized in the Supplementary Table S7. By comparison, total levels of ceramides were comparable between NC and FP/FC group (Supplemental Table S7). By means of individual ceramides with different length of their fatty acid moieties differences could also be observed, especially between NC and FP (Supplemental Table S7). Thus, ceramides C16:0, C18:0 and C26:0 were elevated in blood of FP compared to NC, while reduced levels of ceramides C22:0 and C24:0 were found FP when compared to NC. Consistent with the published results^26^ we found ceramides C16:0, C22:0, C24:0 and C24:1 to be the most abundant among all ceramide species in blood.

It should be noted, however, that the literature data on blood ceramide levels are most often obtained from plasma and serum, whereas we measured DBS extracts, and this, for the first time, without enrichment or derivatization. Therefore, a direct correlation of individual ceramides levels in healthy controls with literature data is rather difficult. While the concentration of some ceramides species like C16:0 or C24:1 are in agreement with literature, other values reveal concentrations lower than plasma or serum values reported previously in healthy individuals^28, 29^.

From all analyzed ceramides, only the concentration of C26:0 ceramide showed a clear-cut difference between the FP (with a value of 61.8 ± 21.8 µg/L blood) and the NC (33.1 ± 7.8 µg/L blood) cohorts with a specificity and sensitivity of 100% (p-value < 0.0001) (Table S7). As shown in the Table S7, JIA patients revealed an overall decrease in all ceramides levels, when compared with FP, FC and NC groups.

Further findings on the screening of ceramides derivatives can be summarized as follows: (i) the total concentration of dihydroceramides was decreased in JIA patients, but comparable between the other three groups; (ii) glucosylceramides levels were clearly increased in the FP group compared with the other three groups, but with a high level of variation within the groups; (iii) the NC group revealed higher levels of sphingomyelins (particularly sphingomyelin C24:0) when compared with FD (patients and carriers) and JIA groups, (iv) the intergroup comparison of other derivatives revealed no certain trends towards increase or decrease of concentrations.

### Ceramide C26

**Figure 1:**
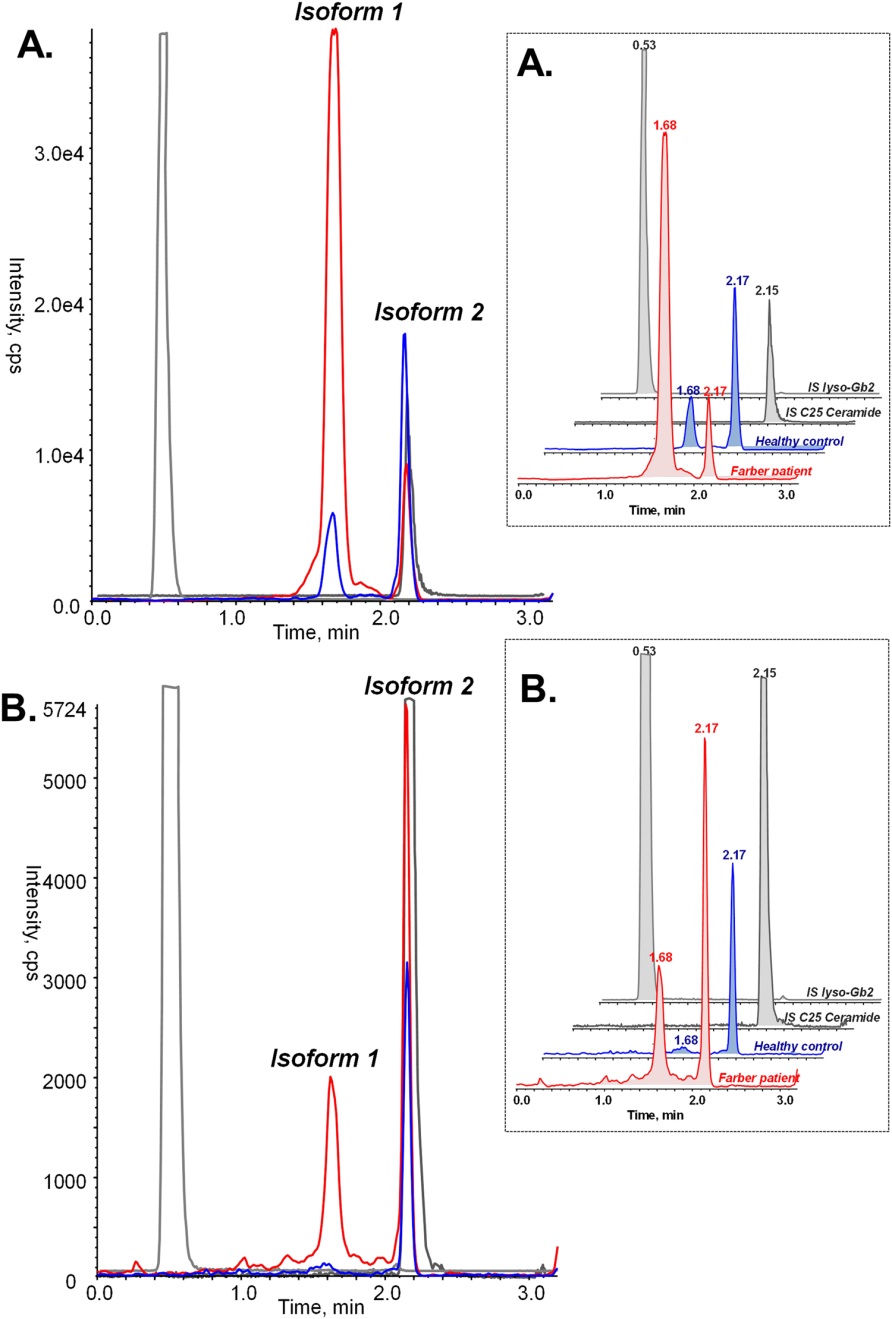
TIC (total ion chromatogram) profile of C26-ceramide isoforms in samples from genetically confirmed Farber patient vs normal control: (**A**)-in DBS; (**B**)-in clear plasma. In DBS, both isoforms were present in both samples from healthy controls (blue) and Farber patients (red), the two internal standards are represented in grey. Isoform 1 was dominant in the Farber samples, while isoform 2 was dominant in healthy controls.

**Figure 2:**
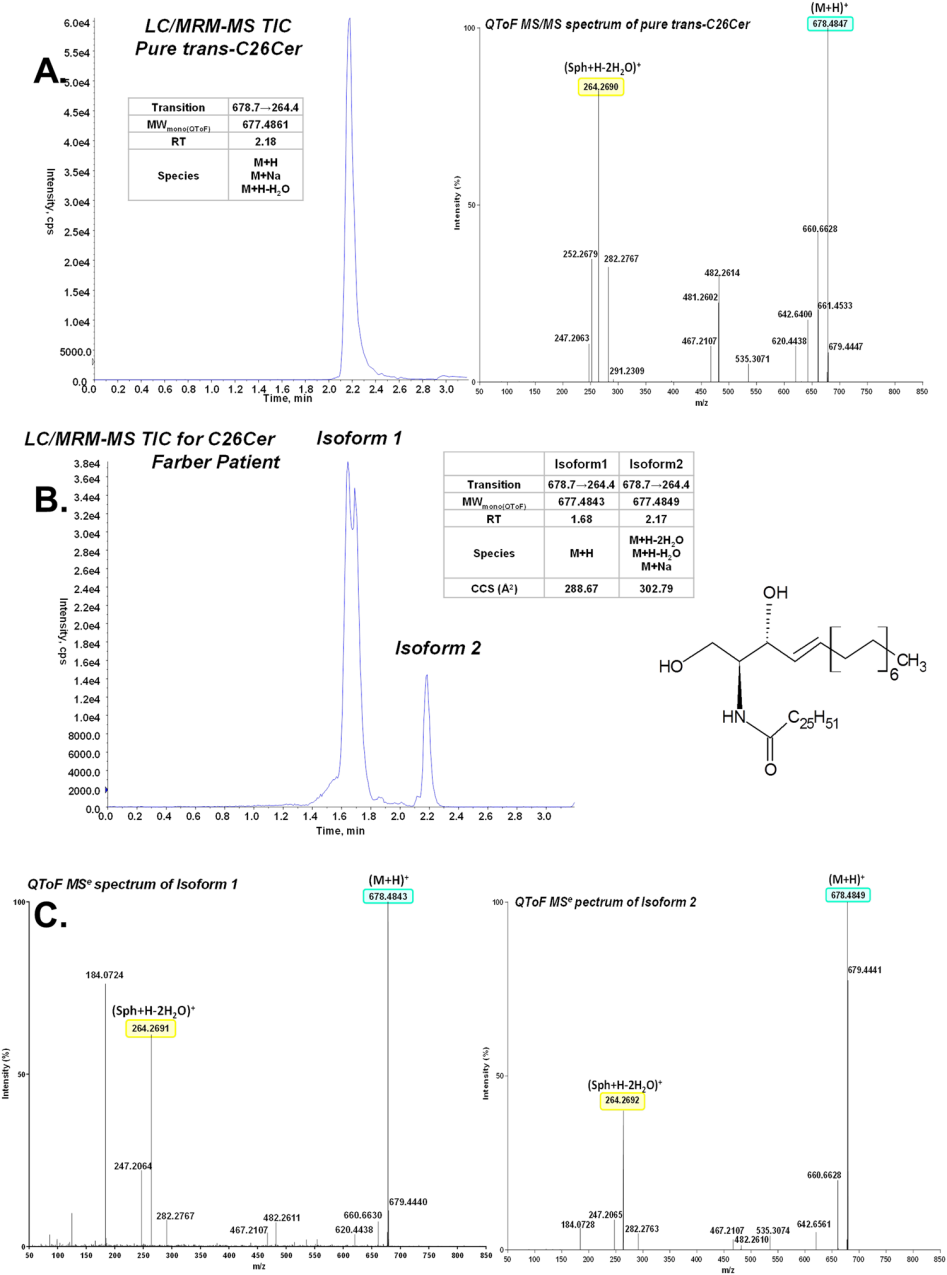
Investigations of the two isoforms of C26:0 ceramide found in DBS extracts: (**A**)-TIC of a LC/MRM-MS analysis and high resolution MS/MS of pure *trans*-C26:0 ceramide; (**B**)-LC/MRM_MS of C26:0 ceramides in DBS extract from a Farber patient and LC/IM-MSe characteristics of the two C26:0 ceramides isoforms. **C**-MS^e^ mass spectrum of the C26:0 ceramide isoforms in DBS.

The overall results of the screening experiments suggested ceramide C26 could be a specific biomarker for FD. Using a commercially available erythro-C26 ceramide (Carbosynth, Canada), the LC-MRM-MS method was further refined to a chromatographic run of 3.1 minutes with improved MRM detection for the monitoring of the C26:0 ceramide and internal standards. During the development and validation of the C26:0 ceramide assay, two internal standards were used: lyso-Gb2 and C25:0 ceramide. We observed instability of C25:0 ceramide solution in ethanol for prolonged periods of time in comparison with lyso-Gb2; C25:0 ceramide was integrated in the study to monitor the quality of the chromatography (the resolution of the chromatographic peak), and lyso-Gb2 was used for the quantification of the C26:0 ceramides. Retention time for the pure *trans*-C26:0 ceramide using this method was found to be 2.17 minutes (Figs 1 and 2). In the biological samples (DBS and plasma extracts) a second peak with the same transition (678.7 → 264.4) was found with a retention time of 1.68 minutes (Fig. 1). The two isoforms were present in all samples from both FD patients and normal controls with different ratios in DBS and plasma. In DBS (Fig. 1A), isoform 1 was dominant in the patient samples while isoform 2 dominated in the control samples. In plasma (Fig. 1B), isoform 1 could be quantified in the patient sample and it was missing in the clear plasma of control samples. These results led to the conclusion that not only the total ceramide C26:0 levels are important in Farber diagnostic, but also the individual isoform concentrations can be used as a diagnostic parameter.

High resolution mass spectrometry (IM-QToF) revealed that the two isoforms have the same molecular mass (m/z 678.4847), and MS^e^ and MS/MS (Fig. 2 and Supplemental Fig. S1) analyses performed on the blood extract confirmed the presence of compounds with similar fragmentation pattern. Correlation of this fragmentation pattern with the pattern of the pure *erythro*-C26 ceramide (Carbosynth,Canada) positively confirmed the presence of both isoforms (Fig. 2). Moreover, ion mobility-mass spectrometry (IM-MS) determined that collision cross section (CCS) of the two isoforms in an untargeted IM-MS^e^ analysis of the dried blood extract is different for the two isoforms, being 288.67 Å^2^ for isoform 1 and 302.79 Å^2^ for isoform 2 (Fig. 2). However, by comparison of spectra between pure *erythro*-C26 ceramide (Fig. 2A) and those obtained from DBS (Fig. 2C), it became obvious, that those are not completely identical. First, there was a fragment 184.07 in DBS samples, eluting ubiquitously from min 1.0 to 2.7, suggesting different types of sphingomyelins are eluting over the gradient in MS^e^ analysis. This fragment was not present in DBS extracts examined by QToF MS/MS method (data not shown). Secondly, another fragment, 252.27 seen in pure *erythro*-C26 ceramide (Fig. 2A), exhibited a much lower intensity (<3% of the highest peak in the spectrum) in DBS extracts in MS^e^ analysis, which can be explained by the lower concentration of the substance in DBS (the calculated concentration was ca 1000 lower in the blood extract compared with the pure substance).

Although in-source degradation of a C26 derivate cannot be completely excluded, our scans did not detect a derivate of C26:0 ceramide that elutes at the same time with isoform 1. We propose here that the two peaks, the isoform 1 and isoform 2 of the C26:0 ceramide, correspond to *cis* and *trans* isomers, respectively. Following facts support our hypothesis: (i) pure *trans*-C26-ceramide has the same retention time (2.17 min) and fragmentation pattern as the isoform 2 found in biological samples (ii) both isoforms have same monoisotopic mass, (iii) both isoforms include fragments of C26:0 ceramide in MS/MS analyses and (iv) the isoforms have different CCS as determined by IM-MS. Our hypothesis needs a further confirmation, such as a chemical synthesis of a *cis*-C26-ceramide and different chemical reactions testing *cis* double bound vs. *trans* double bound. Other potential isomeric forms (e.g. double bond isomerism) cannot be completely ruled out within the present study. Therefore we refer to the C26 isoforms as isoform 1 and isoform 2, both of them uniquely identified by same exact mass and fragmentation pattern, and different retention times and CCSs.

### Normal and pathological range of C26 ceramides

The normal range of C26 ceramides levels in blood was determined on a group of 192 NC. The histograms of the normal distribution of the NC values are added in the supplementary material (Supplemental Fig. S2).

The levels of total ceramide C26 and its isomers obtained in NC were compared to those measured in FP and FC groups (Table 2). In the NC group the total C26:0 ceramide concentration had a value of 49.8 ± 9.6 nmol/L, from which 18.5 ± 4.9 nmol/L was isoform 1 and 33.8 ± 8.6 nmol/L isoform 2. For the pathological range, the FP group (n = 10) was found to have a total C26:0 ceramide concentration of 134.8 ± 52.2 nmol/L blood, from which 94.6 ± 55.4 nmol/L was isoform 1 and 40.1 ± 19.6 nmol/L isoform 2. The cut-off for total C26:0 ceramide was set at 69.0 nmol/L and for isoform 1 at 28.3 nmol/L (mean value of the NC + 2 * standard deviation).

**Table 2:** Concentration of total C26:0 ceramide and its isoforms in the blood of NC, FP and FC.

Samples	N	Ceramide C26:0 (nmol/L) (mean ± STD)					
		Isoform 1	*p-value	Isoform 2	ratio	Total	*p-value
**NC**							
Newborns (0–6 months)	31	18.3 ± 5.8	—	35.3 ± 11.8	0.6 ± 0.3	53.6 ± 11.7	—
Juvenile (0.5–4 years)	78	19.7 ± 5.1	—	25.9 ± 10.43	0.6 ± 0.2	50.5 ± 10.7	—
Adults (>17 years)	83	17.4 ± 4.1	—	31.5 ± 6.5	0.6 ± 0.2	48.6 ± 8.1	—
All NC	192	**18.5 ± 4.9**	—	**33.8 ± 8.6**	0.6 ± 0.2	**49.8 ± 9.6**	—
**FP**							
Newborns (0–6 months)	2	187.5 ± 6.4	0.0038	31.0 ± 2.0	6.1 ± 0.2	218.5 ± 9.1	0.0038
Juvenile (0.5–4 years)	6	80.8 ± 27.1	<0.0001	32.5 ± 9.4	2.6 ± 1.0	113.5 ± 34.05	<0.0001
Adults (>17 years)	2	43.0 ± 12.7	0.0006	72.0 ± 21.2	0.6	115.0 ± 33.9	0.0006
All FP	10	**94.6 ± 55.4**	<0.0001	**40.1 ± 19.6**	2.9 ± 2.0	**134.8 ± 52.2**	<0.0001
**FC**							
Newborns (0–6 months)	1	34.0	n.a.	28.0	1.2	62.0	n.a.
Juvenile (0.5–4 years)	2	193.0 ± 36.0	0.0006	53.0 ± 33.9	4.3 ± 2.1	246.5 ± 71.4	0.0006
Adults (>17 years)	8	59.63 ± 31.89	<0.0001	40.4 ± 13.0	1.6 ± 0.7	99.9 ± 34.1	<0.0001
All FC	11	**81.6 ± 62.8**	<0.0001	**41.6 ± 16.7**	2.0 ± 1.4	**123.1 ± 71.92**	<0.0001
Cut-off (meancontrols + 2 * STD)		28.3		—	—	69.0	

The values are shown as mean ± standard deviation. *p-value found in Mann-Whitney test for FP vs. NC of the same category. NC = normal controls, FP = Farber patients, FC = Farber carriers.

**Figure 3:**
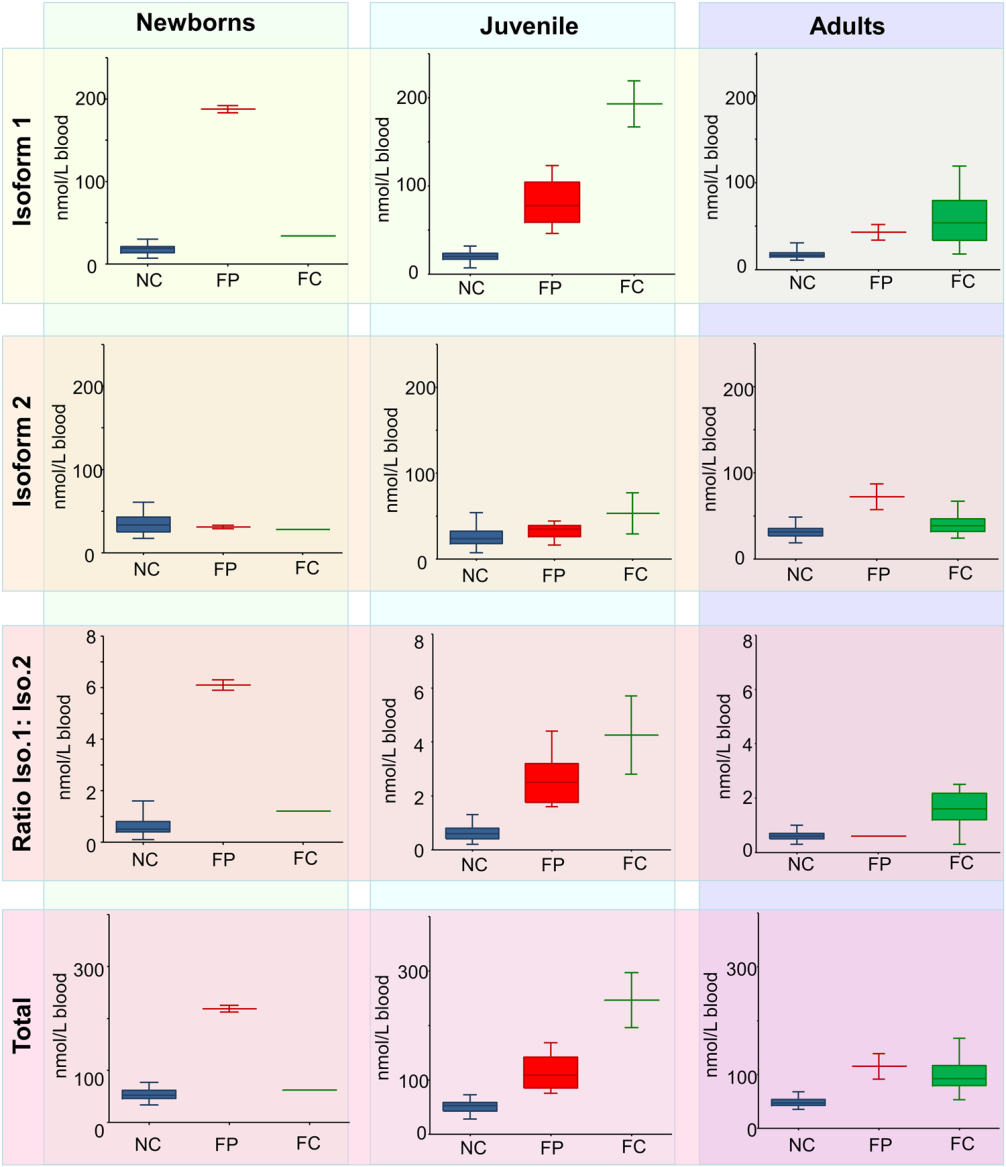
Box plots demonstrating C26:0 ceramide levels in the blood of NC, FP and FC, grouped according to the donors’ ages in newborns (up to 6 months), juveniles (6 months to 17 years) and adults (>17 years). NC = healthy controls, FP = Farber patients, FC = Farber carriers.

When the results of C26:0 ceramide analyses were grouped according to the age into newborns (up to 6 months), juveniles (6 months to 17 years) and adults (>17 years), several trends emerged (Fig. 3).

In NC the concentration ranges of the C26:0 ceramide and its isoforms were independent of age. However, in FP the abundance of the C26:0 ceramide and its isoforms changed with age: the total C26:0 ceramide concentration in newborns (218.5 ± 9.1 nmol/L) was almost twice as high as that in juvenile (113.5 ± 34.05) and adult (115.0 ± 33.9) patients. Furthermore, isoform 1 was extremely high in the newborn FP (187.5 ± 6.4 nmol/L), while a decrease was seen in juvenile FP (80.8 ± 27.1 nmol/L), and even more in the patients that reached adulthood (43.0 ± 12.7 nmol/L, although still above the normal range). The adult FP group also contained SMAMPE-patients who carry the same *ASAH1* mutations as FD patients.

In contrast, the isoform 2 was within the normal range in newborn and juvenile FP (31.0 ± 2.0 and 32.5 ± 9.4 nmol/L, respectively), and increased to pathological levels in FP who reach adulthood (72.0 ± 21.2 nmol/L) (Table 2, Fig. 3).

In FC both isoforms of C26:0 exhibited normal levels in newborn carriers, increasing, however, to pathological levels in juveniles (especially isoform 1). Interestingly, in adults the concentrations of both isoforms were decreased compared to juveniles, but still higher than values seen NC adults (Fig. 3).

Taken together, these results (Table 2, Fig. 3) clearly demonstrate elevated levels of C26:0 in every age group of FP. Quantitative data show that this increase was predominantly caused by alterations of isoform 1, making it, along with total C26:0, to specific biomarker for diagnosis of FD.

### Validation of the C26:0 ceramide test method

Subsequent to the optimization of the quantification of C26:0 ceramide, a full validation of the method was performed including: inter-intra-assay precision, intra-inter assay accuracy, linearity of the measurements, normal range, pathological range, sensitivity and specificity, limit of detection and limit of quantification, robustness, matrix effect and recovery, stability of C26:0 in patient sample over a period of 6 months, limitations of the assay (Table 3).

**Table 3:** Quantification of C26:0 ceramides in dried blood spots - summary of the assay characteristics.

Validation of method		Results	
Accuracy (*trans*-C26Cer) CV (%)	Intra-assay	1.7–3.9	
	Inter-assay	0.3–3.1	
Precision CV (%)	Intra-assay	Isoform 1	1.8–5.5
		Isoform 2	5.6–6.6
		Total	3.3–5.4
	Inter-assay	Isoform 1	4.9–7.1
		Isoform 2	4.3–6.2
		Total	4.8–5.1
Linearity of the measurements		0–200 ng/ml	
Normal range (n = 192) nmol/L		Isoform 1	6.9–27.9
		Isoform 2	3.5–60.7
		Total	28.1–69.7
Reference value (mean + 2STD)		Isoform 1	<27.7 nmol/L
		Total	<69.1 nmol/L
Pathological range (n = 11) nmol/L		Isoform 1	33.7–191.9
		Isoform 2	16.1–87.2
		Total	75.0–224.5
Sensitivity for the tested samples		100%	
Specificity Farber cases vs normal controls (using calculated reference value)		98,9%	
Specificity of the method through LC/MRM-MS		Isoform 1	678.7/264.3 RT: 1.68
		Isoform 2	678.7/264.3 RT: 2.17
Limit of detection (total C26Ceramide)		0.2 nmol/L	
Limit of quantification(total C26Ceramide)		0.7 nmol/L	
Robustness (parameter: extraction time)		40 min vs. 60 min showed no significant difference	
Extraction recovery filter paper		Active blood	40%
		Inactive blood	73%
Matrix effects/recovery from filterpaper (blood spiked with trans-C26Cer) Average n = 10	active blood	40%	
	de-activated blood*	72%	
Stability of indigenous C26 Ceramides		>6 months	

De-activated blood samples were prepared as described elsewhere^55^.

Accuracy of the quantification method was investigated using 7 solutions of *trans*-C26 ceramide of different concentrations, covering different areas of the calibration line (low, medium, high and above the highest point in the calibration line) 6 times on the same batch in 2 different batches. For accuracy, within-run CV% (coefficient of variation) was found to be 1.7 to 3.9% and between-runs 0.3–3.1%.

The precision of the C26 assay was checked during the validation process by determining the C26 ceramide levels in one FP sample and in one NC sample 6 times in the same batch (intra-assay) and in two different batches (inter-assay). The CV% showed no significant differences and remained below 8% (Table 3).

A standard curve was always generated using freshly prepared C26 dilutions in the same solvent mix as the samples. Linearity of the standard curve was determined 5 times in the same batch using 8 solutions of *trans*-C26 ceramide of different concentrations (0 ng/mL to 200 ng/mL). The curve was found to be linear with R values between 0.9981 and 0.9996. The selectivity of the measurement was insured by using LC-MRM-MS, cross-contamination was checked for each batch by injecting IS solution after the highest concentration of the standard curve. Limit of detection (LOD) was found to be 0.2 nmol/L, and limit of quantification (LOQ) 0.7 nmol/L in blank filter paper. Robustness of the method was proved by changing the time for the incubation during the extraction from 40 to 50 and 60 minutes. The results showed no significant difference by increasing the extraction time.

Matrix effect was studied using *trans*-C26 ceramide spiked blood samples to a concentration of 100 ng/mL and 1000 ng/mL. Not-spiked blood samples were used as baseline (to subtract the indigenous C26 ceramide). Each sample was measured 10 times. Blood samples with intact enzyme activity showed low recovery rate (40%) due to the activity of ceramidases during the sample preparation and drying. Blood sample without enzymatic activity showed an average recovery rate of 73%.

In order to demonstrate the stability of both isoforms in DBS extracts, one patient samle was measured 14 times over a period of 6 months.

The only limitation of the assay in plasma was the degree of hemolysis which had a strong influence on the C26:0 ceramide isoforms. Thus, we conclude that the measurements are only reliable when using DBS extract or clear plasma (without traces of hemolysis).

For this limited cohort, the total C26:0 ceramide and isoform 1 levels were increased above cut-off for all the investigated Farber samples, indicating 100% sensitivity. The specificity of the total C26:0 ceramide and isoform 1 as biomarkers for FD was investigated using 10 FP, 192 NC, 2 JIA patients, and 30 patients with genetically confirmed lysosomal storage diseases (LSD), as described in the methods.

**Figure 4:**
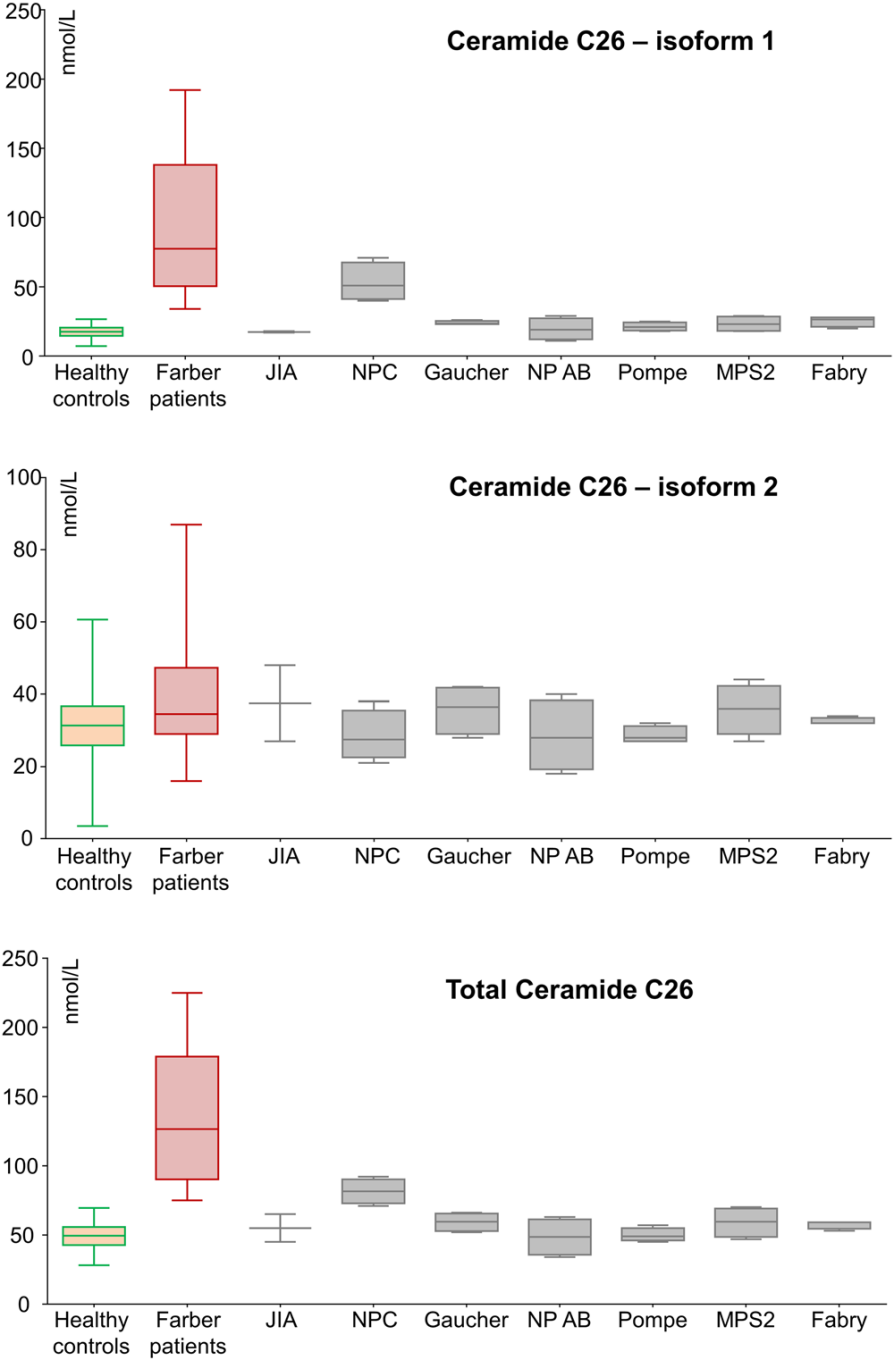
Box plots demonstrating levels of total C26:0 ceramide and its isoforms in the blood of NC, FP, JIA and patients affected by various LSDs. NC = heathy controls, FP = Farber patients, FC = Farber carriers.

The results (Fig. 4, Table 4) show that both biomarkers (total C26:0 ceramide and isoform 1) were increased above the cut-off for the FP (69.0 and 28.3 for total C26 ceramide and isoform 1, respectively). When compared to other pathological conditions, the levels of total C26 and the isoform 1 were significantly higher in the FP group, with the exception of Niemann Pick type C (NPC) disease. Here, the patients exhibited levels of total C26:0 ceramide and isoform 1 clearly above the cut-off.

**Table 4:** Quantification of total C26 ceramide and its isoforms in NC group and different pathological entities.

Samples	N	Ceramide C26:0 (nmol/L)					
		Isoform1	*p-value	Isoform2	Ratio	Total	*p-value
NC	192	18.5 ± 4.9	<0.0001	33.8 ± 8.6	0.6 ± 0.2	49.8 ± 9.6	<0.0001
FP	10	94.6 ± 55.4	—	40.1 ± 19.6	2.9 ± 2.0	134.8 ± 52.2	—
Juvenile idiopathic arthritis	2	17.5 ± 0.7	0.030	37.5 ± 14.8	0.5 ± 0.3	55.0 ± 14.1	0.030
Niemann-Pick Type C disease	5	53.3 ± 13.8	0.142	28.5 ± 7.0	2.1 ± 1.0	81.5 ± 9.037	0.036
Gaucher Disease	5	24.0 ± 1.4	0.002	35.6 ± 6.8	0.7 ± 0.2	59.3 ± 6.8	0.002
Niemann-Pick Type AB disease	5	19.5 ± 7.8	0.002	28.5 ± 9.9	0.8 ± 0.3	48.5 ± 13.3	0.002
Pompe disease	5	21.3 ± 3.0	0.002	28.8 ± 2.3	0.8 ± 0.1	50.0 ± 5.0	0.002
MPS2 disease	5	23.3 ± 5.6	0.002	35.8 ± 7.0	0.7 ± 0.1	59.0 ± 10.8	0.002
Fabry disease	5	25.5 ± 3.8	0.002	32.5 ± 1.0	0.8 ± 0.2	57.5 ± 3.0	0.002
*Cut*-*off*		28.3	—	—	—	69.0	—

*p-value found in Mann-Whitney test for FP vs. affected patients.

It should be mentioned, however, that the concentrations obtained in NPC patients for total C26:0 ceramide (81.5 ± 9.037) were significantly lower (p = 0.036) than those for the FP group (134.8 ± 52.2). As for isoform 1, the difference between FP and NPC groups (94.6 ± 55.4 and 53.3 ± 13.8, respectively) did not reach statistical significance (p = 0.142).

## Discussion

Once considered inert structural components of the plasma membrane, sphingolipids are now acknowledged as biologically active molecules which play a crucial role in regulation of cellular events like cell survival, proliferation, differentiation, growth and inflammatory and apoptotic responses^30^. The backbones of all sphingolipids are ceramides, generated from sphingosine and fatty acids of differing chain-lengths. Numerous pathological conditions are associated with altered levels of ceramides and their metabolizing enzymes. This includes, but is not limited to, diabetes, cancer, infectious lung diseases, Alzheimer’s disease, atherosclerosis, alcoholic liver disease, depression and others^31^. Among these, a particularly aggressive pathologic condition represents Farber disease (FD), an extremely rare autosomal recessive LSD, in which deficiency in acid ceramidase gene leads to excessive ceramide accumulation in the brain, visceral organs, skin, and lymph nodes. The severe neonatal type causes death within the first few days after birth, whereas the mild type lacking neurological involvement has a life expectancy to early adulthood.

Like most LSDs, a clinical suspicion of FD can be biochemically confirmed by quantifying the enzymatic activity in leucocytes or fibroblasts. However, this requires a timely recognition of the disease by the physician and a high amount of material from infant patients. Furthermore, this method is also not suitable for use in high throughput screens. The lack of valid biomarkers for FD motivated us to perform the current study, which, to our knowledge, has the highest number of Farber patients (n = 10) and carriers (n = 11) included to present.

LC-MRM-MS is the technology that can provide the necessary sensitivity, quantitative accuracy and relatively high throughput for sphingolipid analyses in limited sample sizes^32^. This method has considerably extended the clinical view in many pathological conditions, including LSDs. In the present study, we took advantage of the LC-MS/MS to characterize various sphingolipid profiles of FD patients, and patients affected with other LSDs or JIA. For this analysis we used the DBS-method, which, because of obvious advantages, has been increasingly employed in a variety of settings including diagnostic testing, newborns screening, clinical trials, and research for testing enzymes, biomarkers and genetic material.

The most important finding of our study is the identification of a ceramide containing a fatty acid moiety of 26 carbons in length (cerotic acid). As we have shown that the C26:0 is significantly elevated in Farber group, we consider this ceramide to be a biomarker with high sensitivity and specificity.

Most of the previously published human studies on FD report an increased ceramides level in general, not specifying certain fractions of them. In an experimental study conducted on the mouse model of FD, a differential distribution and accumulation of ceramides C16, C24 and C26-ceramide-1-phosphate was demonstrated in kidney medulla and cortex^33^.

According to classical nomenclature cerotic acid is considered as a very long chains fatty acid, VLCFA^34^. Ceramides containing VLCFA areessential components of the permeability barrier in the epidermis^35, 36^. Their deficits have been clearly demonstrated to be involved in skin diseases like psoriasis, atopic dermatitis and certain types of ichthyoses^37^.

The accumulation of ceramide C26 in human subjects, although seldom reported, has beeen linked to different pathologies. Elevated levels of C26 ceramide were found in cerebrospinal fluid of patients with aneurismal subarachnoid hemorrhage^38^. Even though the excess was negligible when compared to other ceramides (C18:0, C24:0, C24:1), the difference to the control group was statistically significant. A study highlighting the importance of ceramides with VLCFA reported that along with C24:0, ceramide C26:0 was the most abundant ceramide in human ischemic left ventricular biopsies, with significantly higher levels compared with C20–C22 ceramides^39^. Elevated levels of C26:0 ceramide were found in diabetic patients with neuropathy, whereas the patients without neuropathy exhibited normal C26 levels comparable to controls^40^. In a mouse model of Zellweger disease, a disorder occurring due to a single peroxisomal protein defect, C26:0 ceramide was demonstrated to accumulate in brain: in addition the C26/C22 ratio showed a 6.5 fold increase in plasma^41^. C26:0 accumulation has been also implicated in other peroxisomal disorders, like neonatal adrenoleukodystrophy and rhizomelic chondrodysplasia punctatae^42^. In general, the available literature shows that C26:0 ceramide accumulates in conjunction with other cermaides containing VLCFA. In contrast, in FD soley the C26:0 level increases and it is not accompanied by an accumulation of other ceramides containing VLCFA.

A possible explanation may be that the mutant acidic ceramidase has a lower substrate activity in hydrolysis of this particular ceramide. Previously, acidic ceramidase substrate specificity was shown to be negatively correlated with fatty acid chain length of synthetic substrate in normal fibroblasts and fibroblasts from patients with FD^43^. Further studies are needed to elucidate the etiology and biological relevance of C26:0 ceramide accumulation in FD. To note, the role of C26:0 ceramide in mammals has not yet been addressed or explored. In yeasts, it has been shown to promote cell apoptosis^44^. Furthermore, C24–C26 ceramides have been shown to mediate the death of a Caenorhabditis elegans mutant that fails to withstand asphyxia, whereas ceramides with shorter chains had the opposite effect^45^.

Notably we also found elevated levels of C26 ceramide in Niemann Pick disease type C (NPC, a neurodegenerative LSD caused by loss-of-function mutations in either the NPC1 or NPC2 genes, both encoding transporter proteins essential for the export of cholesterol from lysosomes)^46^. In NPC, ceramide accumulation occurs mainly because of increased activity of glucosylceramide-synthase in a cholesterol-dependent manner^47^, and reduction of the activity of glucocerebrosidase, another enzyme which hydrolyzes glucosylceramide^48^. Previous literature has also reported elevated levels of glucosylceramide in spleen and liver of NPC patients. Interestingly, in our study we found significantly elevated levels of glucosylceramide species in FP, which is in good agreement with literature data, occurring either secondarily as a consequence of ceramidase deficiency, or due to a deficiency of sphingolipid activator proteins^49, 50^. Despite the similarities described above, the C26:0 level in FD is significantly higher than the one found in NPC. Additionally, the clinical pictures of the two disorders are different.

Reportedly, clinical presentation FD can be misdiagnosed as JIA^24, 25^. This can have very serious consequences as the completely different pathobiologies necessitate different clinical care. We therefore included patients with JIA in our study to compare sphingolipid metabolic profiles between both conditions. Although only present with a small number of patients, the JIA group exhibited ceramide profiles completely different from FD. Particularly, JIA patients demonstrated C26 levels much lower than FD or even the control group, which again supports the consideration of C26 as a biomarker for FD.

In summary, we have demonstrated that LC-MS/MS-based sphingolipid-profiling provides a powerful tool to develop quantifiable measures for disease diagnosis and for assessment of therapy outcomes. The high specificity and sensitivity of Cermaide C26 as a biomarker for FD constitutes a significant advance for the diagnosis and care of Faber patients and will allow for it to be routinely used in high throughput screening.

## Methods

### Patients

The study and the protocol have been approved by the Ethical Committee of the University of Rostock (Ethics vote #A2014-0159). Informed consent was obtained from all participants or their legal guardians. All methods were performed in accordance with the guidelines highlighted in the “World Medical Association Declaration of Helsinki – Ethical Principles for Medical Research Involving Human Subjects”.

DBS were prepared from 192 normal control individuals (NC, 83 adults aged 17–65 years; 78 juveniles aged 0.5–17 years; 31 newborns aged <6 months), 11 Farber carriers (FC, aged 4 months to 35 years) and 10 Farber patients (FP, aged 2 months to 22 years). The FP group compromised 8 patients with Farber lipogranulomatosis and 2 with SMAMPE (spinal muscular atrophy associated with progressive myoclonic epilepsy) - a condition also characterized by mutation in the same gene (*ASAH1*) and accumulation of ceramides, resulting however in slightly different clinical presentation.

Additionally, 2 patients with juvenile idiopathic arthritis (JIA), and patients with LSDs were included. The latter group compromised following patients (genetically confirmed for respective mutations): 5 Niemann-Pick type A/B patients, 5 Niemann-Pick type C patients, 5 Gaucher patients, 5 Pompe patients, 5 mucopolysaccharidosis type II (MPS 2) patients and 5 Fabry patients.

All Farber carriers and patients were genetically confirmed for the presence of an *ASAH1* pathological mutation on both alleles.

### Genetic confirmation of FP (and SMAMPE) patients and FC

Genetic analysis was performed with bidirectional Sanger sequencing of the entire coding region and the highly conserved exon-intron splice junctions using exon specific primers. PCR is followed by Shrimp Alkaline phosphatase/exonuclease I treatment; cycle PCR is carried out using BigDye Terminator kit v3.1 (LifeTechnologies) and subsequent ethanol purification. Sequencing was performed using an ABI 3730 xl sequencer. The test has been developed and validated for clinical purposes. The *ASAH1* gene reference sequence for the main transcript is NM_004315.4. As alternative transcripts in the present work ENST00000262097 (*ASAH1a*) and ENST00000314146 (*ASAH1b*) were used.

All patients presented clinical symptoms consistent with at least one of the FD phenotypes. For all mutations not described in HGMD (or other databases), software analyses have been carried out in Alamut using SIFT, PolyPhen2, MutationTaster and Align GVGD. According to the software predictions the detected novel variants can be considered at least as likely pathogenic.

### Chemicals

For the initial screening of the ceramides, standard pure ceramides were obtained from AVANTI Polar Lipids Inc. (Alabaster, AL, USA) and from Carbosynth Limited (Berkshire, UK). All ceramides were bought with high purity (>99%) and as *trans*-isomer (see supplementary information). As internal standards, non-naturally occurring *trans*-ceramide C25:0 (CerC25:0) and *trans*-ceramide C17:0 were obtained from AVANTI Polar Lipids Inc. (Alabaster, AL, USA, purity 99%) and lyso-lactosylceramide from Biotrend Chemikalien GmbH (Cologne, Germany, purity 98%). For the Ceramide C26:0 biomarker studies, *trans*-C26 ceramide was purchased from Carbosynth Limited (Berkshire, UK). All the LC/MS grade solvents used in biomarker extraction from DBS and in the analysis were purchased from VWR (Darmstadt, Germany). Other chemicals, including formic acid were obtained from Sigma Aldrich (Darmstadt, Germany).

### Sample preparation for mass spectrometric analyses

All dried blood spots (DBS) and plasma samples were prepared under identical conditions for patients and normal controls. To obtain DBS, EDTA blood (50 µL) per spot was dropped on filter paper (CentoCard, Centogene, Germany). Upon drying 2 h at room temperature, the cards were stored sealed in plastic foil and kept at room temperature until further processing. Three punches of 3.2 mm in diameter were cut using a DBS puncher (Perkin Elmer LAS, Germany) and placed in a 2.2 mL round bottom tube (Eppendorf, Germany). 50 μL extraction solution (DMSO: water, 1:1) and 100 μL internal standards solution (200 ng/mL lyso-Gb2 and 30 ng/mL C25:0) were added on top of the paper punches. Samples were mixed using a DVX-2500 Multi-tube vortex device at 2500 rpm for 30 seconds and placed in an incubator (Heidolph, Germany) for 30 minutes at 37 °C under agitation at 700 rpm. After incubation, the tubes were sonicated for 10 minutes at maximum power and then the liquid was transferred to a AcroPrep Filter Plate with PTFE membrane (PALL, Germany) placed on a 96 well V-shape bottom plate (VWR, Germany). The samples were filtrated by centrifugation for 5 minutes at 3500 rpm in a Hermle Z300 plate centrifuge (Hermle Labortehnik, Germany) to remove any solid particles from the solution.

For plasma sample preparation, 25 μL of plasma, 100 μL of the same internal standard solution and 250 μL ethanol were mixed into a 1.7 mL tube (Eppendorf, Germany) using a DVX-2500 Multi-tube vortex device at 2500 rpm for about 30 seconds and placed in the refrigerator (Heidolph, Germany) for 60 minutes at 4 °C. After protein precipitation, the tubes were centrifuged for 5 minutes at 14.500 rpm using a Benchtop centrifuge (Eppendorf, Germany) and then 200 μL of supernatant were transferred to the AcroPrep Filter Plate with PTFE membrane. Similar to DBS preparation, also here the samples were filtrated to remove any solid particles from the solution.

### Liquid chromatography- mass spectrometry analyses

#### High resolution mass spectrometry and LC/MRM-MS characterization of ceramides standards

High resolution mass spectrometry analysis of the standards was performed using a Waters i-Class Acquity UPLC (Waters, UK) coupled with a Vion IMS QToF (Waters, UK). Chromatographic run was performed on a C8, 3 µm, Ultra-Inert HPLC Column, 50 × 2.1 mm (ACE, ACE, Germany) using a flow rate of 0.9 mL/min preheated at 60 °C. 10 µL of standard at concentration of 100 ng/mL in ethanol were injected. The analytes were eluted using a gradient with type 6 curve from 40% A (50 mM formic acid in water) to 100% B (50 mM formic acid in acetone: acetonitrile vol. 1:1). MS/MS QToF measurement were performed in positive ion mode, with scan m/z 50–1000, scan time of 1 s, CE ramp 20–80 eV, desolvation temperature of 600 °C, desolvation gas 1000 L/h, source temperature 150 °C, cone gas 50 L/h, capillary coltage 3 kV, mass tolerance 0.002. LC/MRM-MS analysis was performed on a Waters Acquity UPLC (Waters, UK) coupled with an ABSciex 5500 TripleQuad mass spectrometer (ABSciex, Germany). LC parameters were identical with the high resolution. Upstream from UPLC a 3:1 flow splitter was added. MRM-MS analyses were performed in positive ion mode using the following parameters: CUR gas 10 psi, IS voltage 5 kV, CAD 8 psi, cone temperature 200 °C, GS 1 45 psi, GS2 60 psi, EP 10 V.

#### Preliminary LC-MRM-MS ceramide study

At an initial step we developed a method to screen for ceramides and ceramide derivatives with medium and long acyl chains, in order to identify compounds that can be used as a specific biomarker for acidic ceramidase deficiency in FD. For this purpose samples were comprised within 3 groups. First group included adult healthy controls, aged 17–65, a heterogeneous group which included persons with diabetes and obesity as previous studies reported elevated ceramides levels in these two groups. In the second group samples from FP and FC were used. Patients with juvenile idiopathic arthritis (JIA), an autoimmune disorder with similar clinical manifestation as FD, represented the third group. The details of the preliminary study, including the scanned molecules, LC/MRM-Ms parameters, examples of individual ion chromatograms and the results of the ceramide screening are listed in supplementary information (Supplemental Tables S1–S7). Based on the results of the screening we further focused on the ceramide C26:0 and its isoforms.

#### High resolution mass spectrometry analyses of C26 ceramides in DBS extracts

High resolution mass spectrometry analysis and ion mobility analyses were carried on a Waters i-Class Acquity UPLC (Waters, UK) coupled with a Vion IMS QToF (Waters, UK). LC parameters were conserved for all the analyses: 10 µL extract were injected on a C8, 3 µm, Ultra-Inert HPLC Column, 50 × 2.1 mm (ACE,ACE, Germany) and the compounds were eluted using a gradient with type 6 curve from 40% A (50 mM formic acid in water) to 100% B (50 mM formic acid in acetone: acetonitrile vol. 1:1). Ion mobility separation on full scan mass spectrometry (IM-MS^e^) used the following parameters: high definition MSe mode, m/Z 50–1000, scan time 0.5 s, low CE 6 eV, high CE ramp 20–60 eV, desolvation temperature 600 °C, source temperature 150 °C, desolvation gas 1000 L/h, cone gas 50 L/h, Capillary voltage 3 kV, lock correction enabled (lock sprayer reference: mass 556.2766 m/z, interval 1 min, sample time 1 min, CE 18 eV, flow rate 10 µL/min), enabled CCS screen, with tolerance of 2%, expected adducts +H, +K, +Na. Processing of the raw data was performed using a preset precursor ion of 678.484.

#### LC/MRM-MS standardized method for C26 ceramide quantification

LC**/**MRM-MS analyses of the C26:0 ceramide (Supplemental Tables S4 and S8) for both DBS and plasma extracts were performed using a Waters Acquity UPLC (Waters, UK) coupled with an ABSciex 5500 TripleQuad mass spectrometer (ABSciex, Germany). The chromatographic run was performed on a C8, 3 µm, Ultra-Inert HPLC Column, 50 × 2.1 mm (ACE,ACE, Germany) using a flow rate of 0.9 mL/min preheated at 60 °C. The 10 µL extract were injected on the column and the compounds were eluted using an exponential gradient from 40% A (50 mM formic acid in water) to 100% B (50 mM formic acid in acetone: acetonitrile vol. 1:1). Upstream from UPLC a 3:1 flow splitter was added. The following MRM transitions were monitored: 624.3 → 282.2 (with DP 30 V, CE 38.4 V and CXP 13 V) and 664.5 → 264.4 for the internal standards IS1 amd IS2 (DP34V, CE 46 V and CXP 11 V) and 678.7 → 264.4 for C26 ceramide isoforms (with DP of 34 V, CE of 46 V and CXP of 11 V). MRM-MS analyses were performed in positive ion mode using the following parameters: CUR gas 10 psi, IS voltage 5 kV, CAD 8 psi, cone temperature 200 °C, GS 1 45 psi, GS2 60 psi, EP 10 V. For all batches analyzed a standard curve was measured using 9 dilutions of C26 ceramide in ethanol (concentrations in ng/mL: 0; 1; 2; 5; 10; 25; 50; 100; 200) and control samples of a FP and one NC with known concentrations of C26-ceramides. Data analysis was performed using Analyst 1.6.2 and Microsoft Office 2010.

### Statistical analysis

Statistical analyses were carried out using GraphPad Prism 5 data analysis software (GraphPad Software, La Jolla, USA). The Non-parametric Mann-Whitney U test was chosen for the statistical analysis due its robustness and to the limited number of control and Farber samples. Values of p ≤ 0.05 were considered as statistically significant.

## Electronic supplementary material

Supplementary Information
