# Supplementary material for: C26-Ceramide as highly sensitive biomarker for the diagnosis of Farber Disease
Source: Sci Rep. 2017 Jul 21;7:6149. doi: 10.1038/s41598-017-06604-2 (PMC5522391; doi:10.1038/s41598-017-06604-2)
Supplement: Supplementary file 1 — Supplementary Information [file 41598_2017_6604_MOESM1_ESM.pdf]

Supplementary information

Title: C26-Ceramide as highly sensitive biomarker for the diagnosis of Farber Disease

Authors: Claudia Cozma[1*], Marius-Ionuț Iurașcu[1], Sabrina Eichler[1], Marina Hovakimyan[1], Oliver Brandau[1], Susanne Zielke[2], Tobias Böttcher[2], Anne-Katrin Giese[2], Jan Lukas[2], Arndt Rolfs[1,2]

[1]Centogene AG, Schillingallee 68, 18057 Rostock, Germany

[2]Albrecht-Kossel-Institute for Neurodegeneration, Rostock University Medical Centre, Gehlsheimerstraße 20, 18147 Rostock, Germany

[*]Correspondence and requests should be addressed to

claudia.cozma@centogene.com

marina.hovakimyan@ centogene.com

Supplementary Table S1. Standards and internal standards used in ceramide screening in DBS.

No.	Compound	Formula	Monoisotopic mass	Average mass	RT	Fragment ion to be monitored
**Ceramides**						
Std 1	Ceramide C6:0	$C_{24}H_{49}NO_3$	399.3712	399.65	1.3	264.25660
Std 2	Ceramide C12:0	$C_{30}H_{59}NO_3$	481.4495	481.79	1.6	264.26899
Std.3	Ceramide C14:0	$C_{32}H_{63}NO_3$	509.4808	509.84	1.8	264.26899
Std 4	Ceramide C16:0	$C_{34}H_{67}NO_3$	537.5121	537.90	1.9	264.26899
Std 5	Ceramide C24:0	$C_{42}H_{83}NO_3$	649.6373	650.11	2.2	264.26922
Std 6	Ceramide C24:1	$C_{42}H_{81}NO_3$	647.6216	648.09	2.2	264.26899
Std 7	Ceramide C26:0	$C_{44}H_{87}NO_3$	677.6686	678.16	2.2	264.26899
**Dihydroceramides**						
Std 8	Dihydroceramide C12:0	$C_{30}H_{61}NO_3$	483.4651	483.81	1.7	266.28508
Std 9	Dihydroceramide C18:0	$C_{36}H_{73}NO_3$	567.5590	567.96	2.0	266.28460
Std 10	Dihydroceramide C24:0	$C_{42}H_{85}NO_3$	651.6529	652.12	2.2	266.28447
**Glucosylceramides**						
Std 12	Glucosylceramide C16:0	$C_{40}H_{77}NO_8$	699.5649	700.04	1.8	264.3
Std 13	Glucosylceramide C18:0	$C_{42}H_{81}NO_8$	727.5962	728.09	1.9	264.26813
**Sphingomyelins**						
Std 14	Sphingomyelin C12:0	$C_{35}H_{71}N_2O_6P$	646.5050	646.92	1.4	184.07282
Std 15	Sphingomyelin C18:0	$C_{41}H_{83}N_2O_6P$	730.5989	731.08	1.8	184.07280
	**Lactosylceramides**					
Std 16	Lactosylceramide C12:0	$C_{42}H_{79}NO_{13}$	805.5551	806.07	1.6	264.26895
-	**Internal Standards**					
IS1	Lyso-Gb2	$C_{30}H_{57}NO_{12}$	623.3881	623.77	0.5	282.27880
IS2	Ceramide C25:0	$C_{43}H_{85}NO_3$	663.6529	664.1399	2.2	264.26693
IS3	Ceramide C17:0	$C_{35}H_{69}NO_3$	551.5277	551.9273	1.8	264.26862

Supplementary Table S2. High resolution MS/MS spectra and LC/MRM-MS TIC profiles of the standards used in the ceramide screening study (raw data)

High resolution QToF MS/MS spectrum	TIC of LC/MRM-MS analysis
Std1 – Ceramide C6:0	
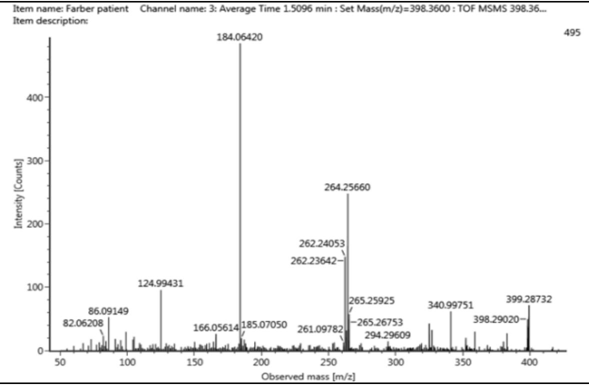	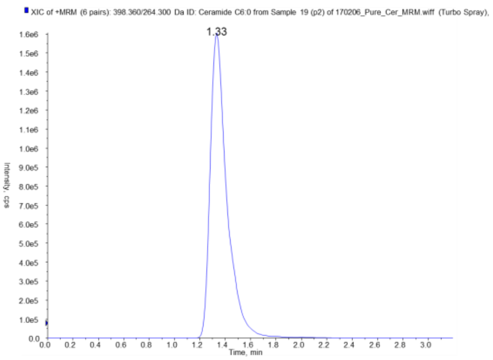
Std2 – Ceramide C12:0	
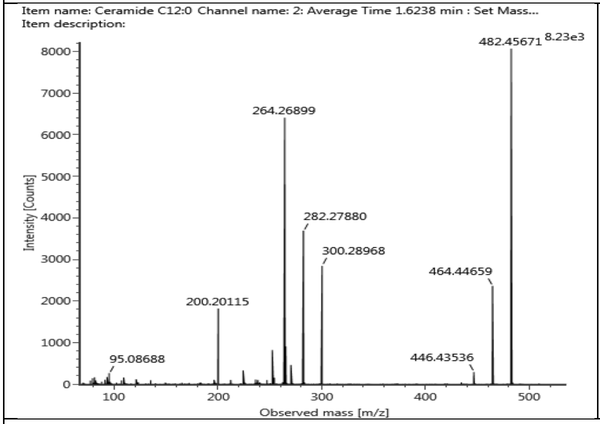	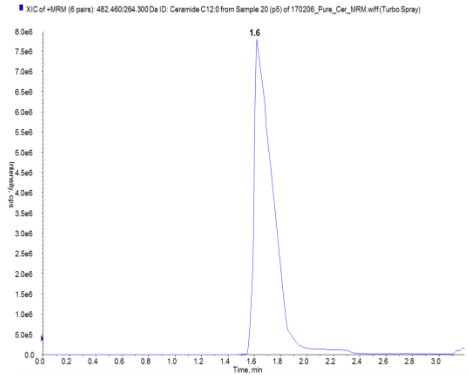
Std3 – Ceramide C14:0	
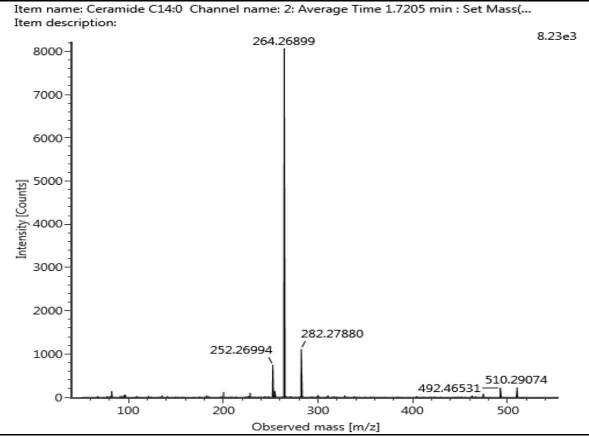	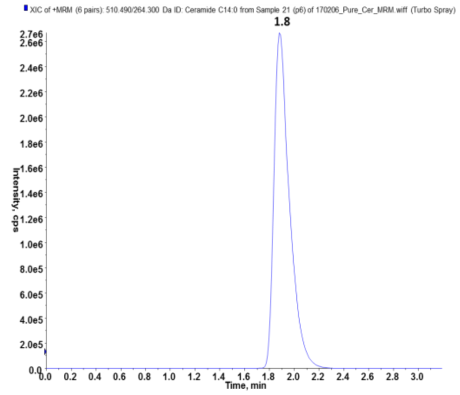
Std4 – Ceramides C16:0	

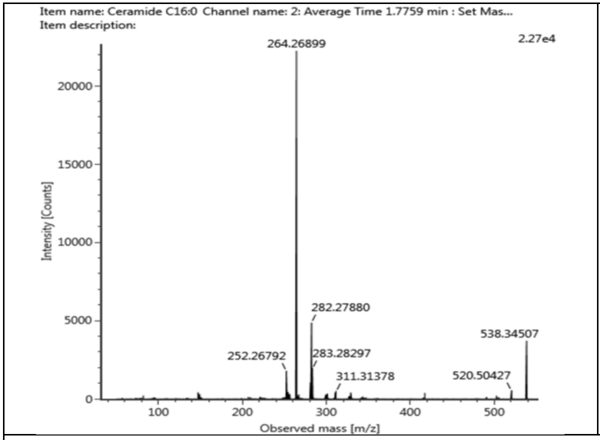

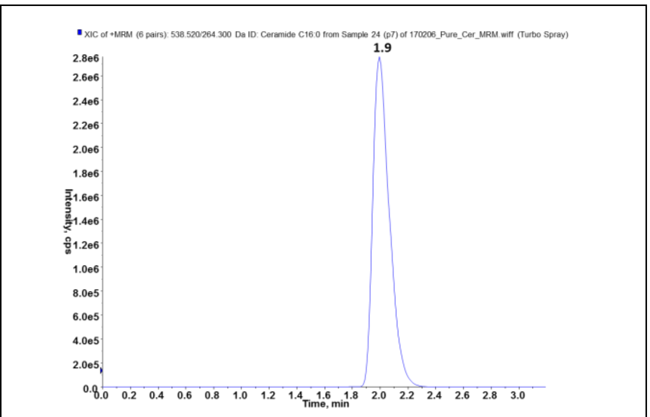

Std 5 – Ceramide C24:0

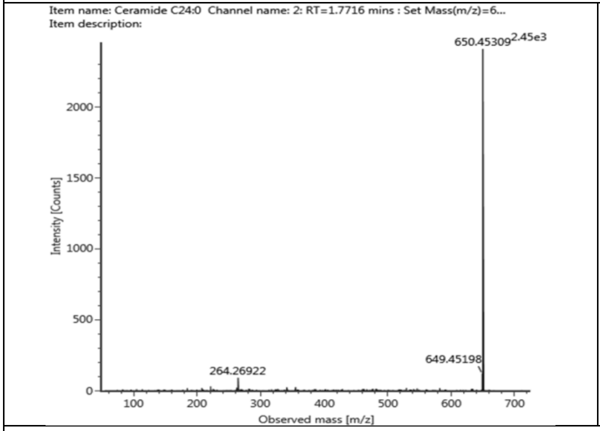

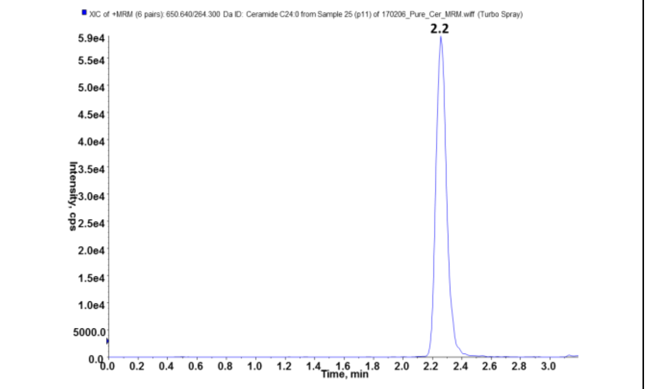

Std 6 – Ceramide 24:1

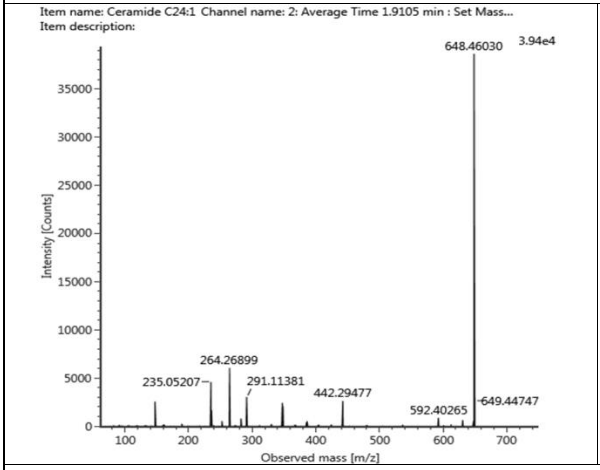

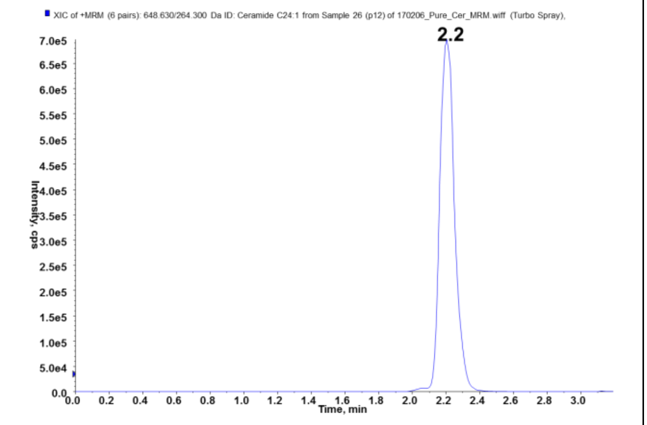

Std 7 – Ceramide C26:0

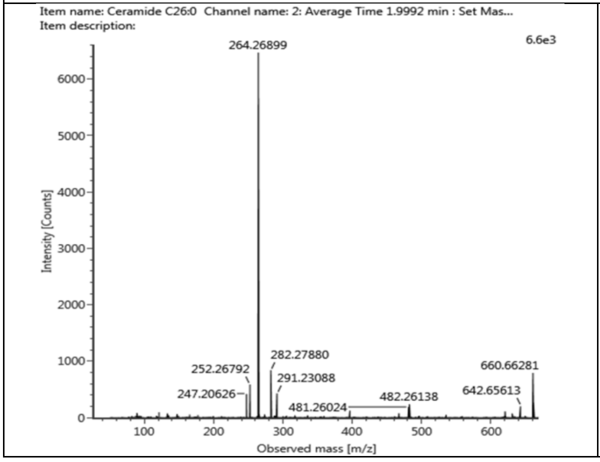

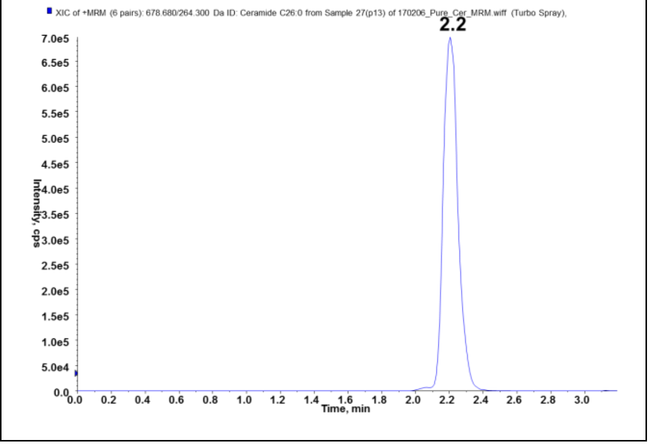

Std 8 – Dihydroceramide C12:0

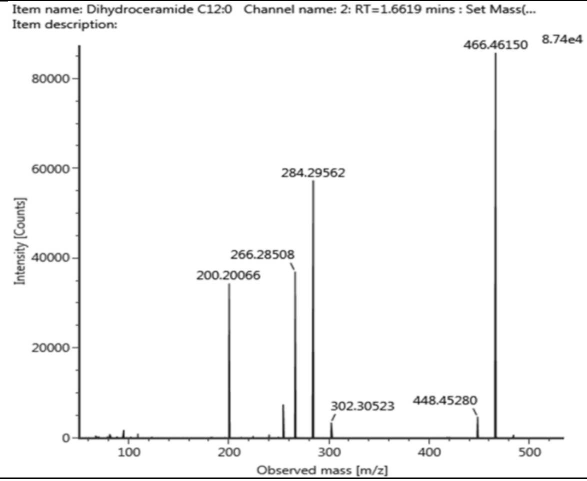

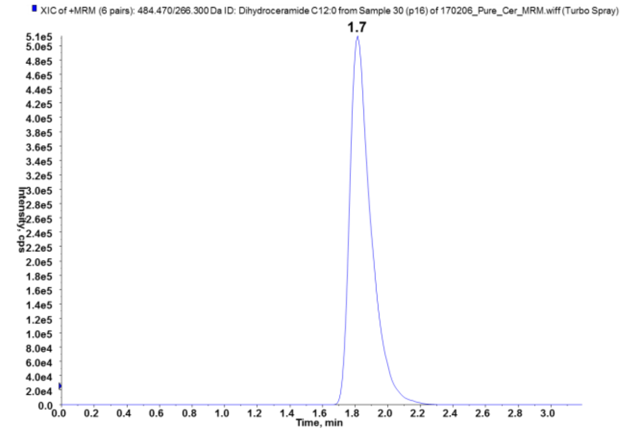

Std 9 – Dihydroceramide C18:0

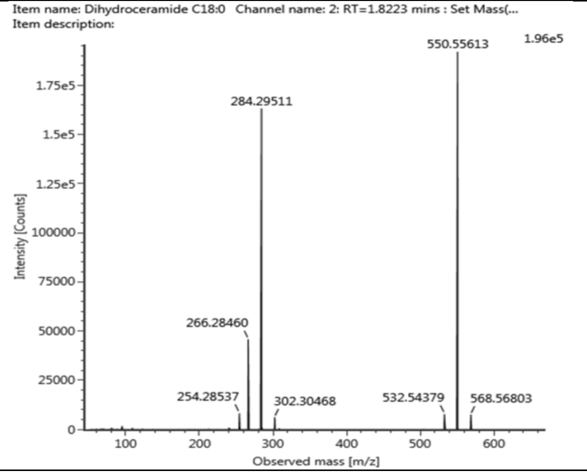

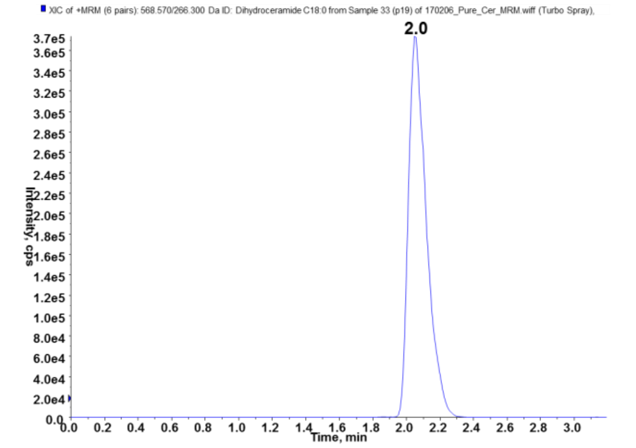

Std 10 – Dihydroceramide C24:0

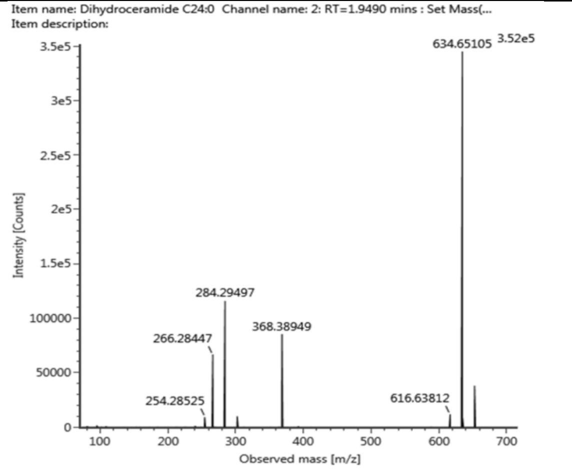

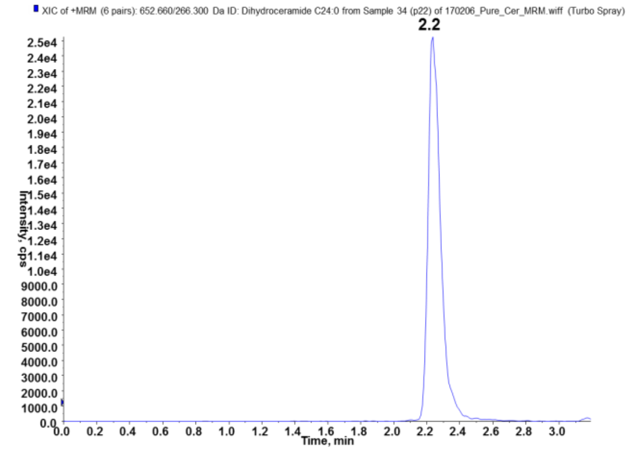

Std 11 – Glucosylceramide C16:0

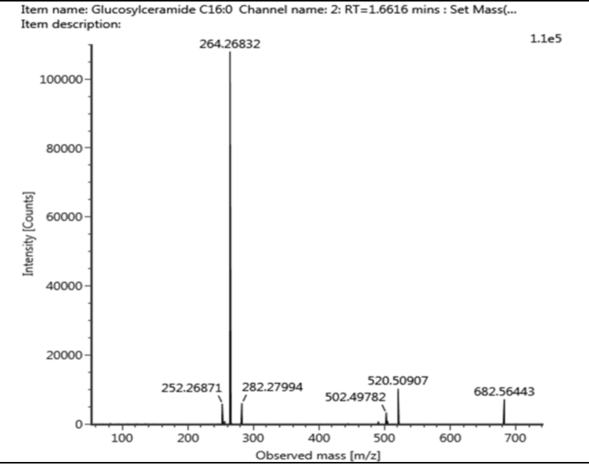

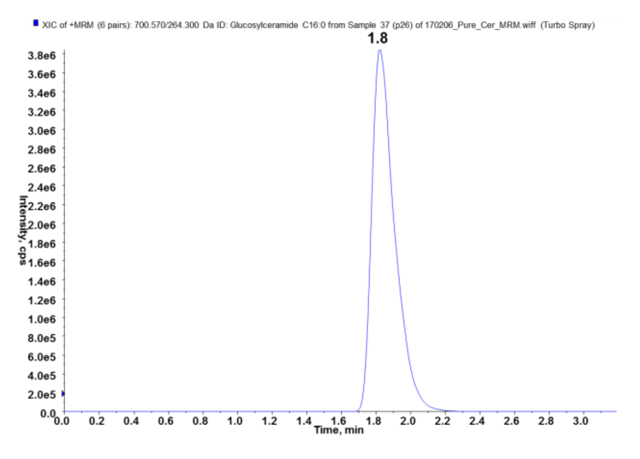

Std 12 – Glucosylceramide C18:0

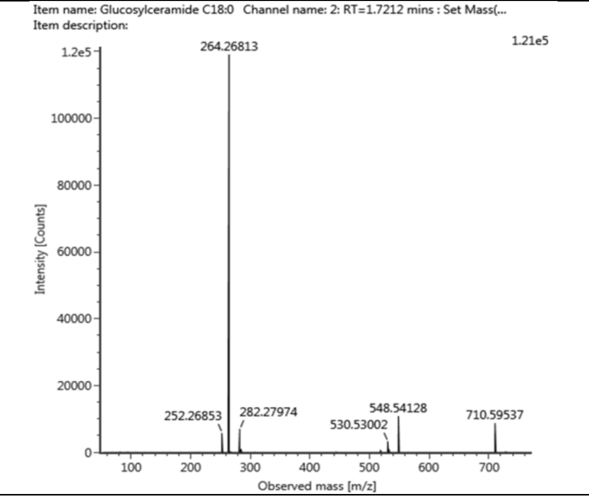

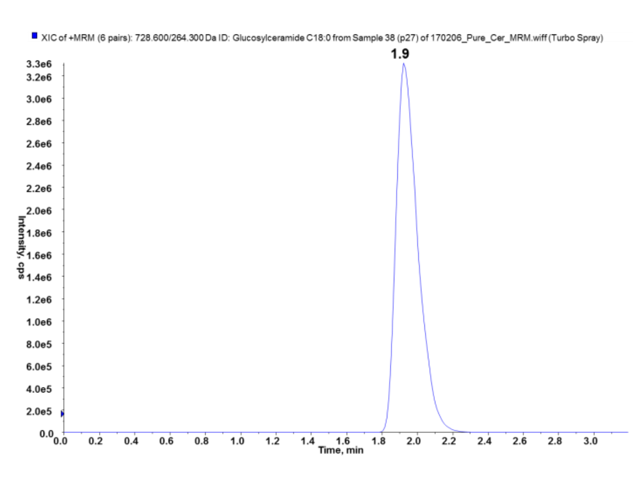

Std 13 Sphingomyelin C12:0

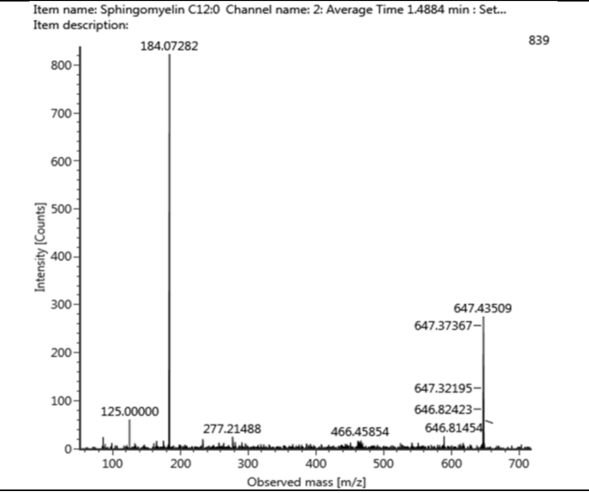

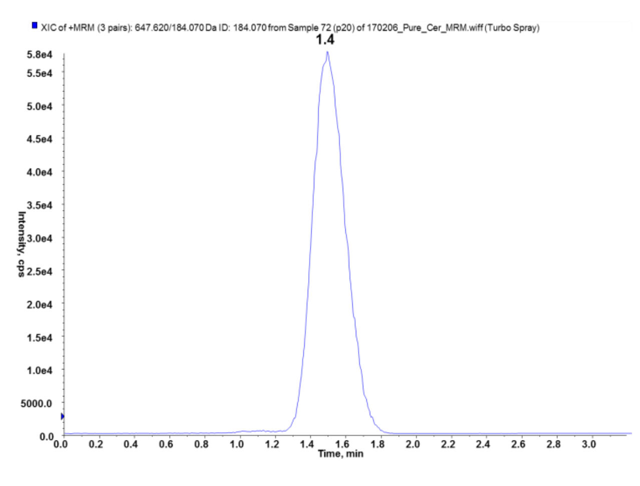

Std 14 Sphingomyelin C18:0

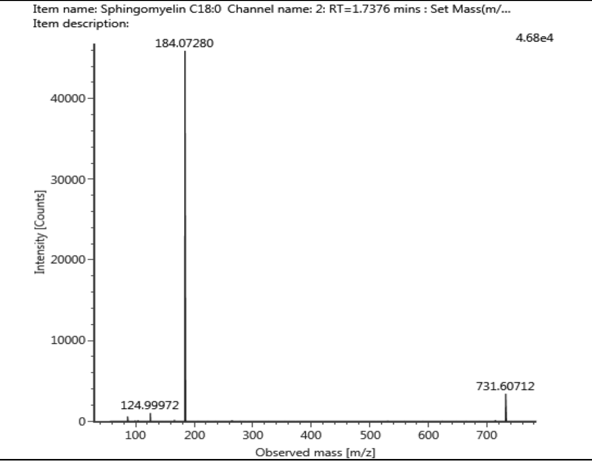

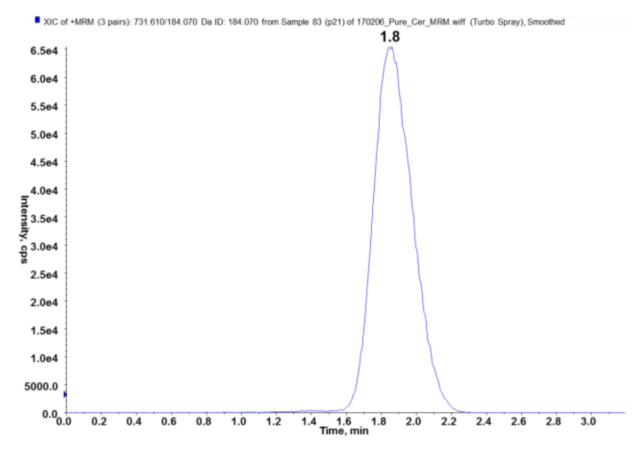

Std 15 - Lactosylceramide C12:0

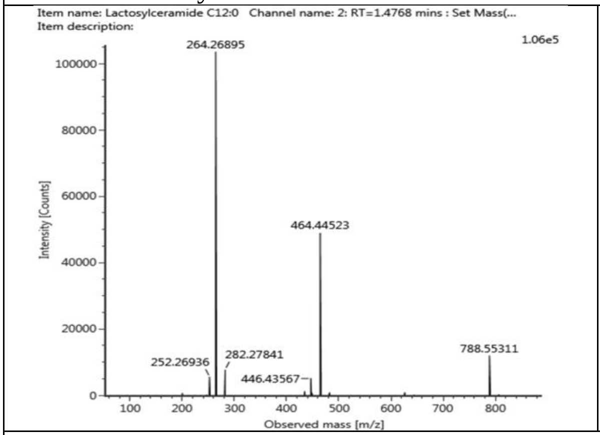

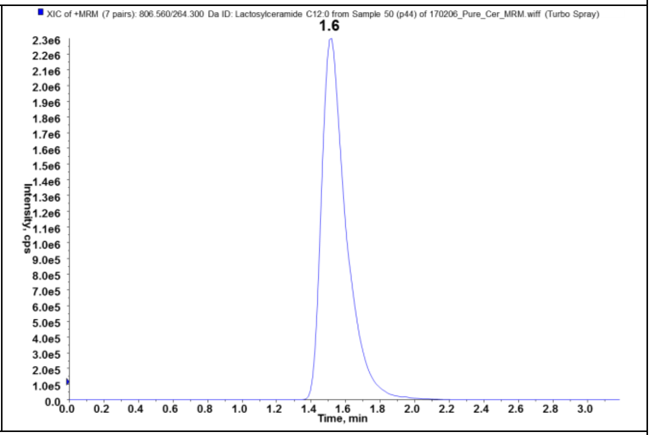

IS 1 – Lyso-lactosylsphingosine (lyso-Gb2)

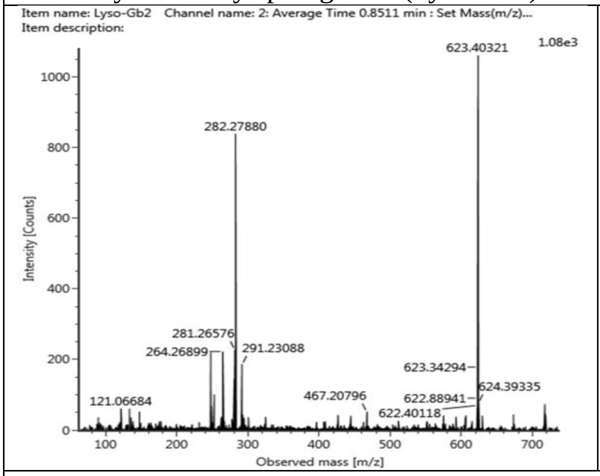

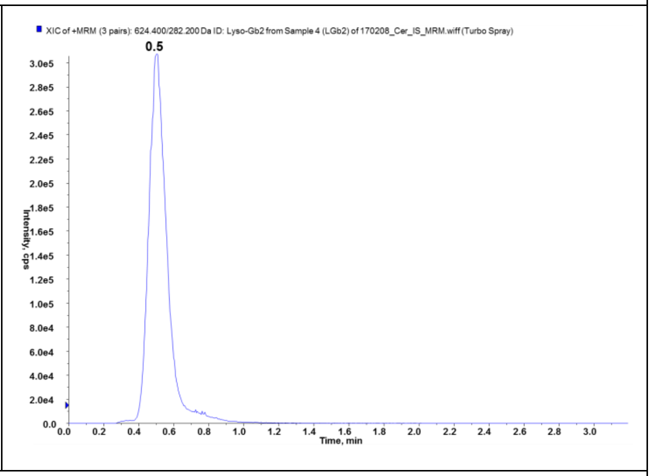

IS2 – Ceramide C25:0

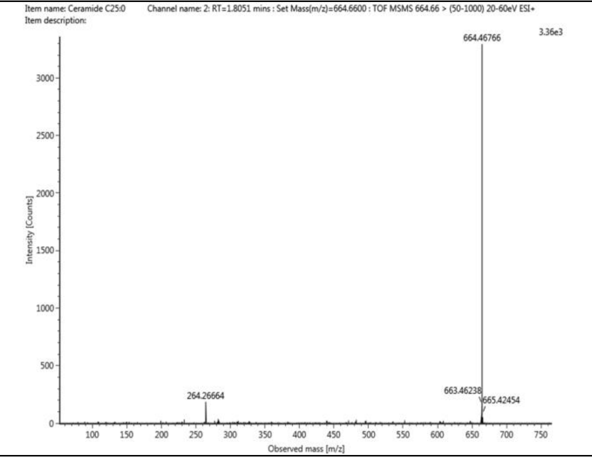

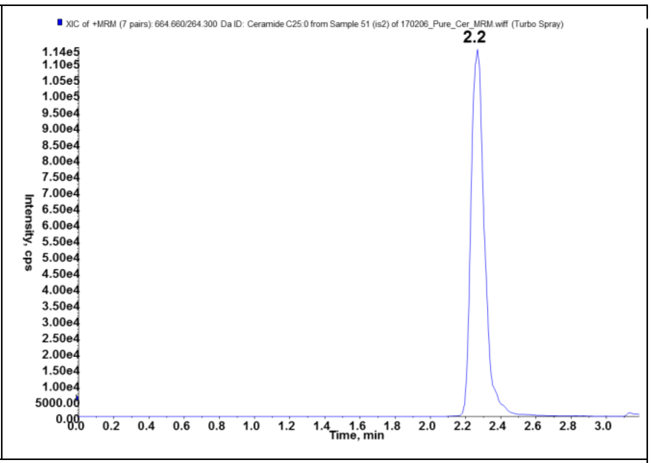

IS3 – Ceramide C17:0

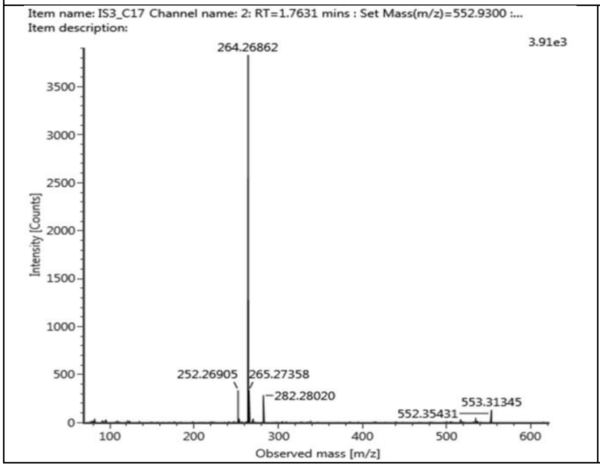

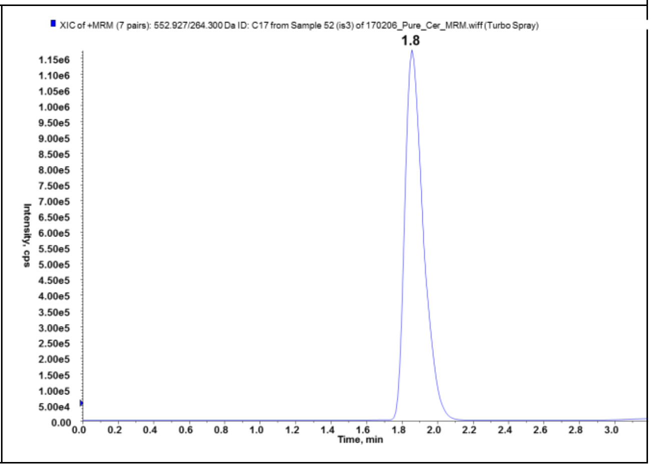

Supplementary Figure S1. Fragmentation pattern of the ceramides

A. Fragmentation pattern observed for the ceramides with a sphingosine base

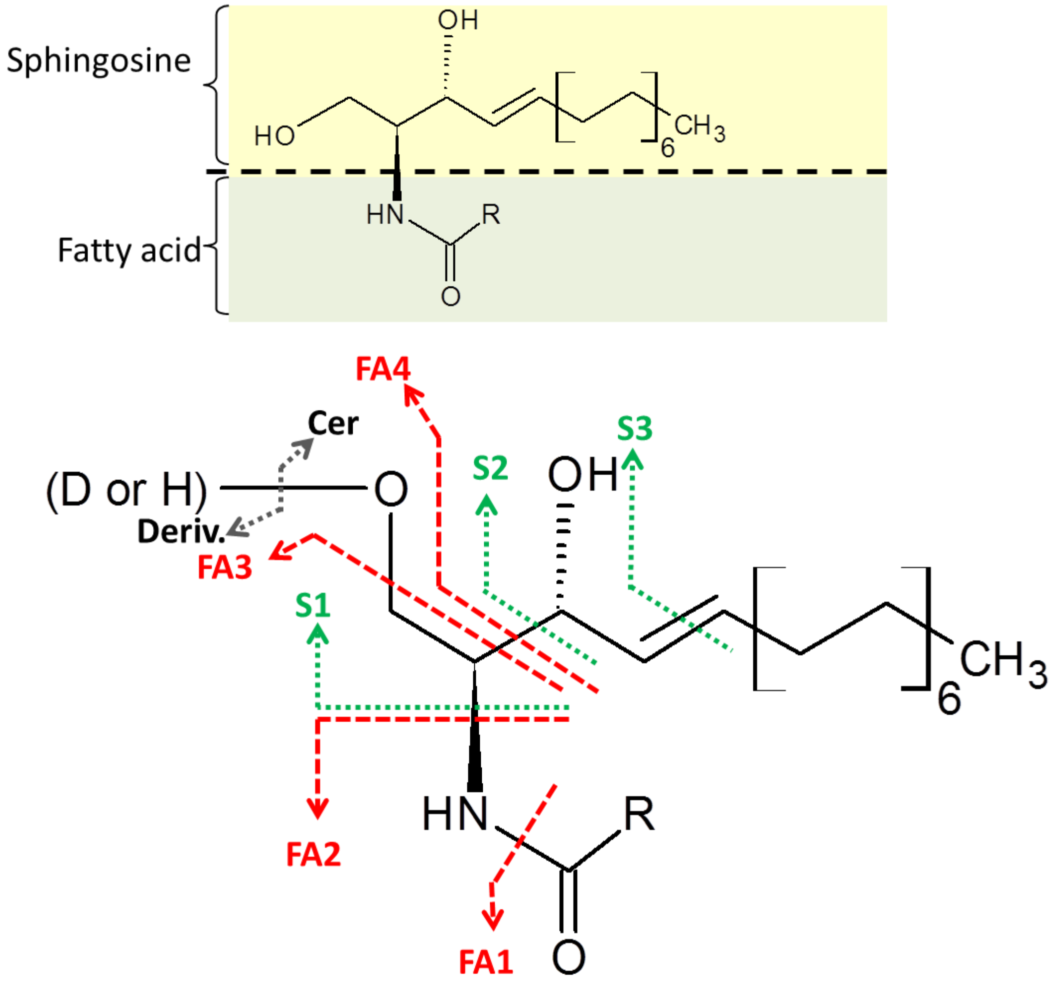

B. Fragmentation pattern observed for the ceramides with a sphinganine base

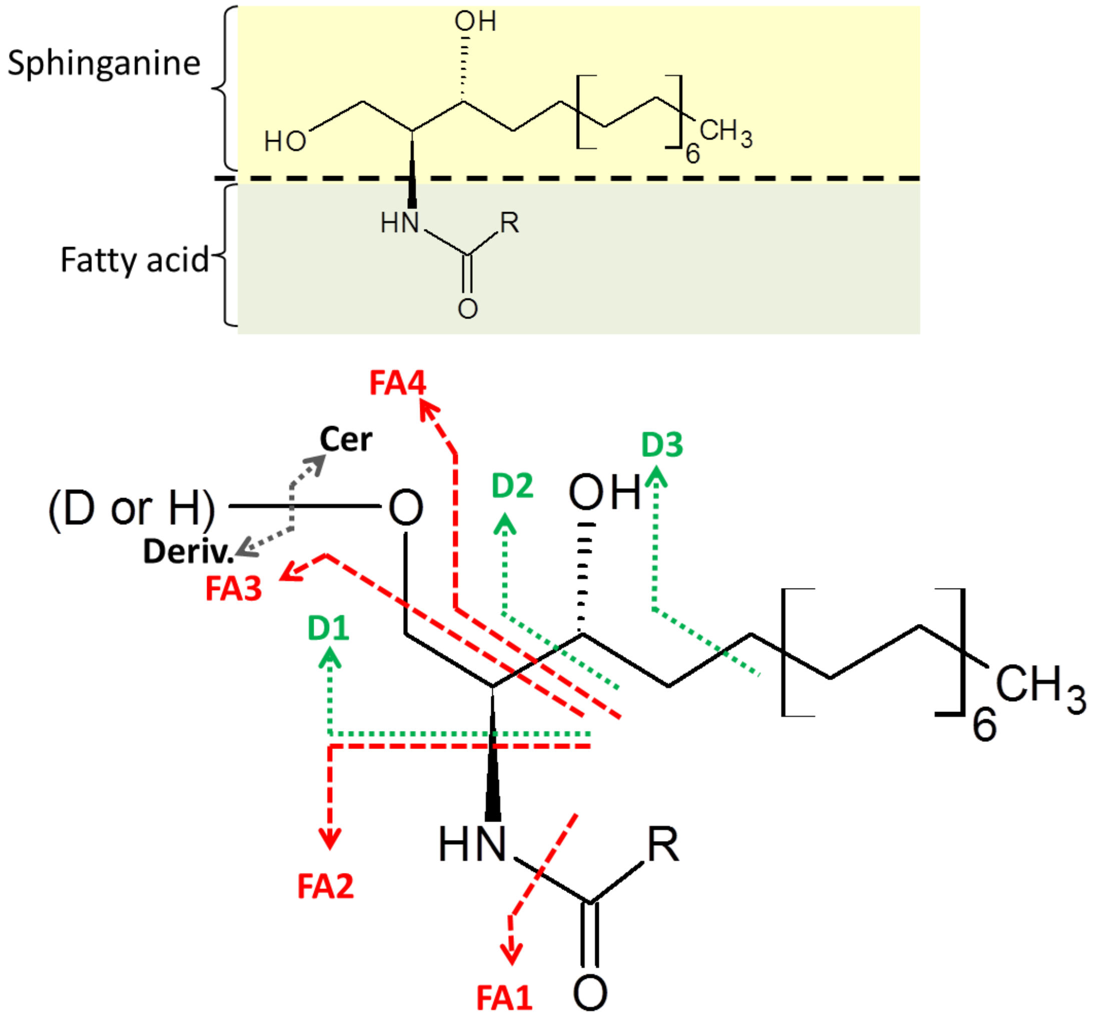

Supplementary Table S3. Parameters for LC/MRM-MS method used for ceramide screening

**LC parameters**								
Instrumentation	Waters UPLC Acquity							
Solvent A	50 mM formic acid							
Solvent B	50 mM formic acid in Acetone:Acetonitrile 1:1							
Column	ACE C8, 3µm, Ultra-Inert HPLC Column, 50x2.1mm (ACE,Germany)							
Flow	0.9 mL/min,							
Flow splitter	upstream to MS ; Waste to MS 2:1							
Initial gradient*	Time(min)	0.0	0.2		6.5	9.4	9.5	10
	% B	60	60		100	100	0	0
Final gradient*	Time(min)	0.0	0.2	1.7	1.8	2,8	2,9	3.1
	% B	60	60	100	100	100	40	40
Gradient curve	6							
Column temperature	60°C							
Equilibration time	0.1 minutes							
**MRM-MS parameters**								
Instrumentation	ABSciex 5500 TripleQuad							
Relative start time	0.0							
Experiment in period	1							
Scan type	MRM							
Polarity	Positive							
Ion source	Turbo spray							
Resolution Q1	unit							
Resolution Q3	unit							
MR Pause	5.000 msec							
CAD	8 psi							
CUR	10 psi							
GS1	45 psi							
GS2	60 psi							
IS	5000 V							
TEM	200 °C							
EP	10 V							
**Transitions monitored**	**See supplementary table S4**							

*Initial gradient was used for detection of the ceramides and ceramide derivatives, subsecvent optimizations let to the final gradient listed in the table.

Note for the quantification method: (i.)for the analytes with available pure synthetic standard, the quantification was performed using standard curves obtained with these standards; (ii.) for analytes with not available pure synthetic standard, the slopes from a standard structurally close was used for quantification (from each class of ceramides or ceramides derivatives , at least one synthetic standards was used).

Supplementary Table S4. Ceramides and ceramides derivatives targeted for ceramide screening in dried blood spots extract

No.	Compound	R	Formula	Monoisotopic mass	LC/MRM-MS parameters				
					RT	Q1 mass (m/z)	Q3 mass (m/z)	DP (V)	CE (V)
	**Ceramides**	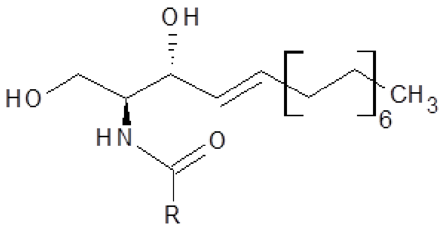							
A1.	Ceramide C4:0	C_3H_7	$C_{22}H_{45}NO_3$	371.3399	1.2	371.8	264.4	34	44
A2.	Ceramide C6:0	C_5H_{11}	$C_{24}H_{49}NO_3$	399.3712	1.2	398.8	264.4	34	44
A3.	Ceramide C8:0	C_7H_{15}	$C_{26}H_{51}NO_3$	425.3869	1.2	426.8	264.4	34	44
A4.	Ceramide C10:0	C_9H_{19}	$C_{28}H_{55}NO_3$	453.4182	1.5	454.8	264.4	34	44
A5.	Ceramide C12:0	$C_{11}H_{23}$	$C_{30}H_{59}NO_3$	481.4495	1.6	482.8	264.4	34	44
A6.	Ceramide C14:0	$C_{13}H_{27}$	$C_{32}H_{63}NO_3$	509.4808	1.8	510.8	264.4	34	45
A7.	Ceramide C16:0	$C_{15}H_{31}$	$C_{34}H_{67}NO_3$	537.5121	1.9	538.8	264.4	34	45
A8.	Ceramide C18:0	$C_{17}H_{35}$	$C_{36}H_{71}NO_3$	565.5434	1.9	566.8	264.4	34	45
A9.	Ceramide C20:0	$C_{19}H_{39}$	$C_{38}H_{75}NO_3$	593.5747	2.0	594.8	264.4	34	45
A10.	Ceramide C22:0	$C_{21}H_{43}$	$C_{40}H_{79}NO_3$	621.6060	2.1	622.8	264.4	34	46
A11.	Ceramide C24:0	$C_{23}H_{47}$	$C_{42}H_{83}NO_3$	649.6373	2.1	650.5	264.4	34	46
A12.	Ceramide C24:1	$C_{23}H_{45}$	$C_{42}H_{81}NO_3$	647.6216	2.2	648.8	264.4	34	46
A13.	Ceramide C26:0	$C_{25}H_{51}$	$C_{44}H_{87}NO_3$	677.6686	1.7 & 2.2	678.7	264.4	34	46
	**Dihydroceramides**	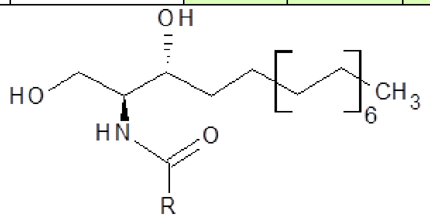							
A14.	Dihydroceramide C8:0	C_7H_{15}	$C_{26}H_{53}NO_3$	427.4025	1.4	428.8	266.4	34	44
A15.	Dihydroceramide C10:0	C_9H_{19}	$C_{28}H_{57}NO_3$	455.4338	1.5	456.8	266.4	34	44
A16.	Dihydroceramide C12:0	$C_{11}H_{23}$	$C_{30}H_{61}NO_3$	483.4651	1.7	484.8	266.4	34	44
A17.	Dihydroceramide C14:0	$C_{13}H_{27}$	$C_{32}H_{65}NO_3$	511.4964	1.8	512.8	266.4	34	45
A18.	Dihydroceramide C16:0	$C_{15}H_{31}$	$C_{34}H_{69}NO_3$	539.5277	1.9	540.8	266.4	34	45
A19.	Dihydroceramide C18:0	$C_{17}H_{35}$	$C_{36}H_{73}NO_3$	567.5590	2.0	568.8	266.4	34	45
A20.	Dihydroceramide C20:0	$C_{19}H_{39}$	$C_{38}H_{77}NO_3$	595.5903	2.0	596.8	266.4	34	45
A21.	Dihydroceramide C22:0	$C_{21}H_{43}$	$C_{40}H_{81}NO_3$	623.6216	2.1	624.8	266.4	34	46
A22.	Dihydroceramide C24:0	$C_{23}H_{47}$	$C_{42}H_{85}NO_3$	651.6529	2.2	652.5	266.4	34	46
A23.	Dihydroceramide C24:1	$C_{23}H_{45}$	$C_{42}H_{83}NO_3$	649.6373	2.2	650.8	266.4	34	46
A24.	Dihydroceramide C26:0	$C_{25}H_{51}$	$C_{44}H_{89}NO_3$	679.6842	2.3	680.5	266.4	34	46

	**Glucosylceramides**	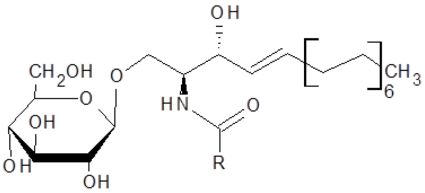							
A25.	Glucosylceramide C12:0	$C_{11}H_{23}$	$C_{36}H_{69}NO_8$	643.5023	1.8	644.6	264.4	34	44
A26.	Glucosylceramide C16:0	$C_{15}H_{31}$	$C_{40}H_{77}NO_8$	699.5649	1.8	700.7	264.4	34	45
A27.	Glucosylceramide C18:0	$C_{17}H_{35}$	$C_{42}H_{81}NO_8$	727.5962	1.9	728.7	264.4	34	45
A28.	Glucosylceramide C24:0	$C_{23}H_{47}$	$C_{48}H_{93}NO_8$	811.6901	2.0	812.9	264.4	34	46
	**Dihydro-glucosylceramides**	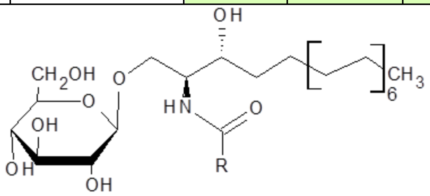							
A29.	Dihydro-glucosylceramide C12:0	$C_{11}H_{23}$	$C_{36}H_{71}NO_8$		1.8	646.6	266.4	34	44
A30.	Dihydro-glucosylceramide C16:0	$C_{15}H_{31}$	$C_{40}H_{79}NO_8$		1.8	702.7	266.4	34	45
A31.	Dihydro-glucosylceramide C18:0	$C_{17}H_{35}$	$C_{42}H_{83}NO_8$		1.8	730.7	266.4	34	45
A32.	Dihydro-glucosylceramide C24:0	$C_{23}H_{47}$	$C_{48}H_{95}NO_8$		1.9	814.9	266.4	34	46
	**Sphingomyelins**	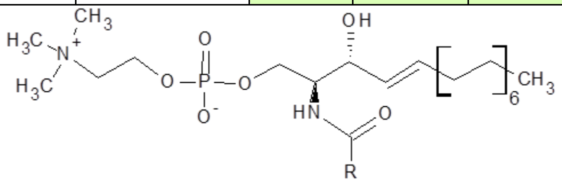							
A33.	Sphingomyelin C12:0	$C_{11}H_{23}$	$C_{35}H_{71}N_2O_6P$		1.4	647.7	184.1	34	44
A34.	Sphingomyelin C18:0	$C_{17}H_{35}$	$C_{41}H_{83}N_2O_6P$		1.6	731.8	184.1	34	45
A35.	Sphingomyelin C24:0	$C_{23}H_{47}$	$C_{45}H_{95}N_2O_6P$		1.8	815.9	184.1	34	46
	**Dihydrosphingomyelins**	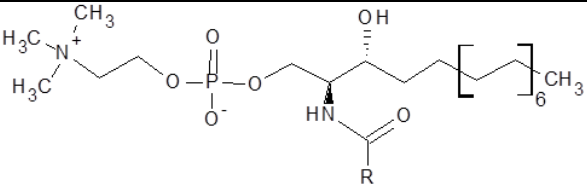							
A36.	Dihydro-sphingomyelin C12:0	$C_{11}H_{23}$	$C_{35}H_{73}N_2O_6P$	648.5206	1.4	649.7	184.1	34	44
A37.	Dihydro-sphingomyelin C18:0	$C_{17}H_{35}$	$C_{41}H_{85}N_2O_6P$	732.6145	1.8	733.8	184.1	34	45
A38.	Dihydro-sphingomyelin C24:0	$C_{23}H_{47}$	$C_{47}H_{97}N_2O_6P$	816.7084	2.1	817.9	184.1	34	46
	**Lactosylceramides**	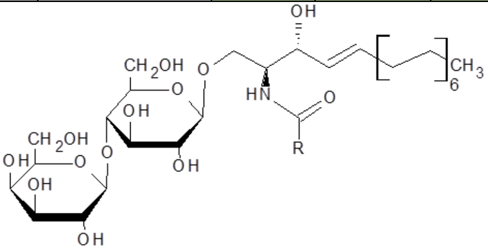							
A39.	Lactosylceramide C12:0	$C_{11}H_{23}$	$C_{42}H_{79}NO_{13}$	806.0756	1.4	806.6	264.4	34	44
A40.	Lactosylceramide C16:0	$C_{15}H_{31}$	$C_{46}H_{87}NO_{13}$	861.6177	1.8	862.7	264.4	34	45
A41.	Lactosylceramide C24:0	$C_{23}H_{47}$	$C_{54}H_{103}NO_{13}$	973.7429	2.0	974.9	264.4	34	46
	**Internal Standards**								
IS1.	Lyso-Gb2	H	$C_{30}H_{57}NO_{12}$	623.7731	0.5	624.3	282.2	30	38.4

IS2.	Ceramide C25:0	$C_{24}H_{49}$	$C_{43}H_{85}NO_3$	663.6529	2.2	664.5	264.4	34	46
IS3.	Ceramide C17:0	$C_{16}H_{33}$	$C_{35}H_{69}NO_3$	551.5277	1.8	552.9	264.4	32	43

Supplementary Table S5. Ion fragments observed in high resolution MS^{e} spectra of the targeted ceramides and ceramides derivatives measured directly in DBS extracts

Ceramide fragments (Cer) for ceramides with Sphingosine base	
**Fragment species**	**Mass modification (monoisotopic)**
$[Cer+H]^+$	+1.0078
$[Cer+H_2O+H]^+$	+19.0232
$[Cer+H-H_2O]^+$	-17.0027
$[Cer+H-2H_2O]^+$	-35.0133
$[Cer+H-H_2O-CH_2O]^+$	-47.0133
**Ceramide fragments (Cer) for ceramides with Sphinganine base**	
**Fragment species**	**Mass modification (monoisotopic)**
$[Cer+H]^+$	+1.0078
$[Cer+H-H_2O]^+$	-17.0027
$[Cer+H-2H_2O]^+$	-35.0133
$[Cer+H-H_2O-CH_2O]^+$	-47.0133
**Fatty acid fragments (FA)***	
**Fragment species**	**Mass modification (monoisotopic)**
$[RCO]^+$	+27.9949
$[RCH_2NO+H]^+$	+45.0215
$[RC_3H_6NO+H]^+$	+73.0527
$[RC_3H_6NO_2+H]^+$	+89.0477
**Sphingosine fragments (S)**	
**Fragment species**	**Fragment mass (monoisotopic)**
$[Sph+H]^+$	300.2903
$[Sph+H_2O+H]^+$	318.3008
$[Sph+H-H_2O]^+$	282.2797
$[Sph+H-2H_2O]^+$	264.2691
$[Sph+H-H_2O-CH_2O]^+$	252.2691
$[Sph+H-C_2H_5NO]^+$	259.2637
$[Sph+H_2O+H- C_2H_5NO]^+$	241.2531
$[Sph+H- C_3H_7NO_2]^+$	229.2531
$[Sph+H_2O+H- C_3H_7NO_2]^+$	211.2426
**Sphinganine fragments (D)**	
**Fragment species**	**Fragment mass (monoisotopic)**
$[DSph+H]^+$	302.3059
$[DSph+H-H_2O]^+$	284.2953
$[DSph+H-2H_2O]^+$	266.2848
$[DSph+H-H_2O-CH_2O]^+$	254.2848
$[DSph+H-C_2H_5NO]^+$	243.2688
$[DSph+H- C_3H_7NO_2]^+$	213.2582
*R = alkyl fragment of the fatty acid listed in Supplementary Table S4	

Supplementary Table S6. TIC profiles of the LC/MRM-MS analysis for the targeted ceramides and ceramides derivatives in Farber extract vs. Controls

Ceramides

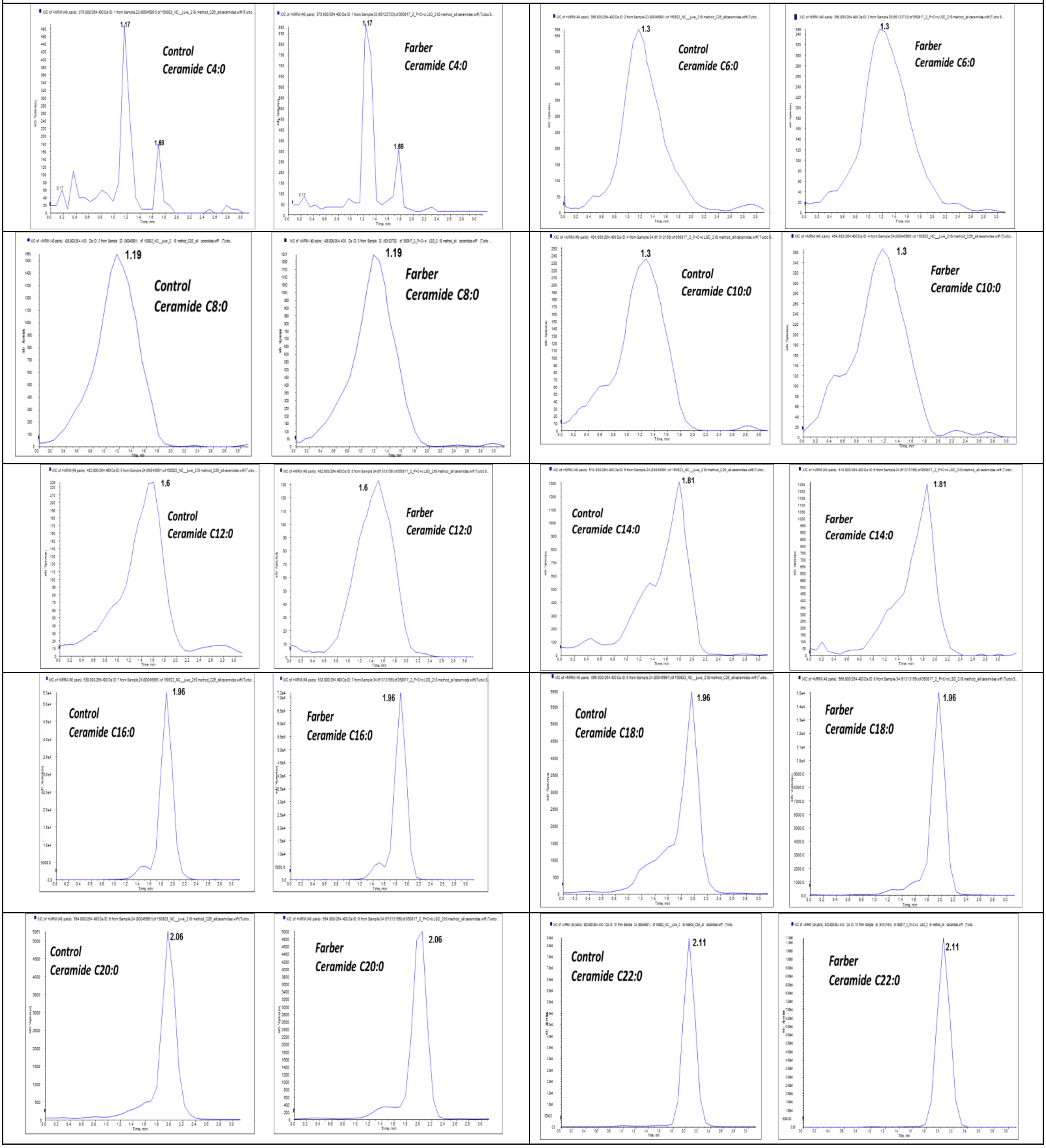

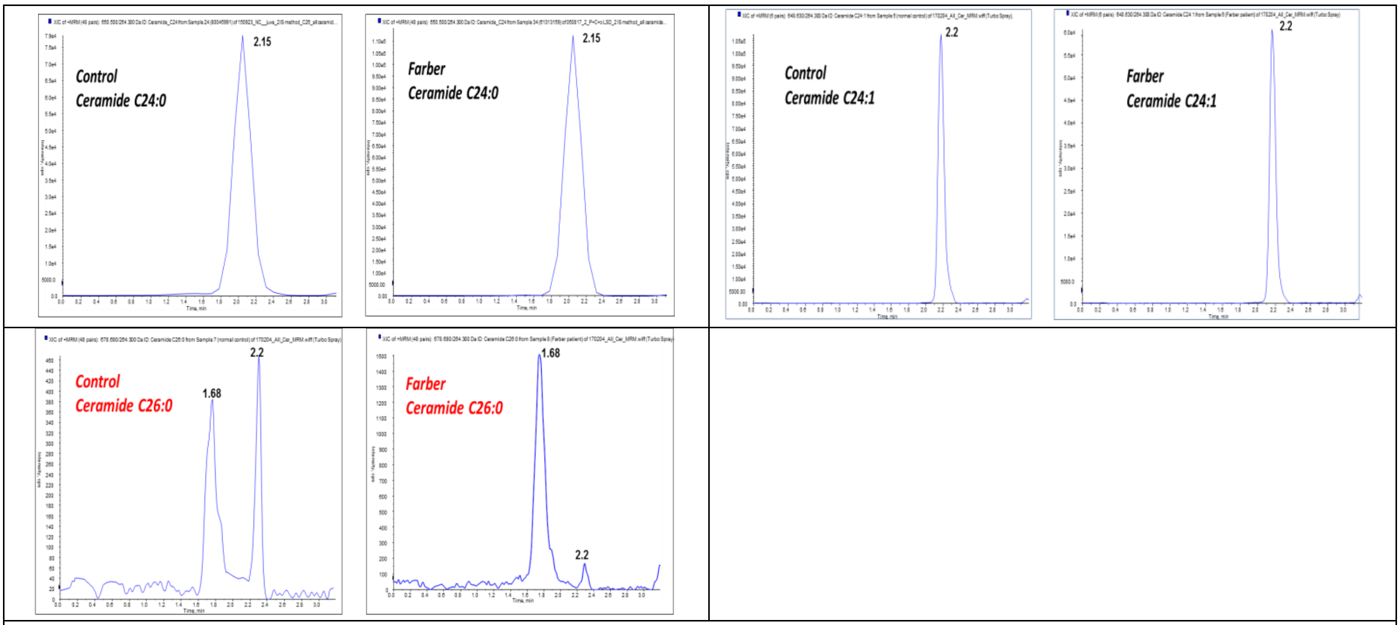

Dihydroceramides

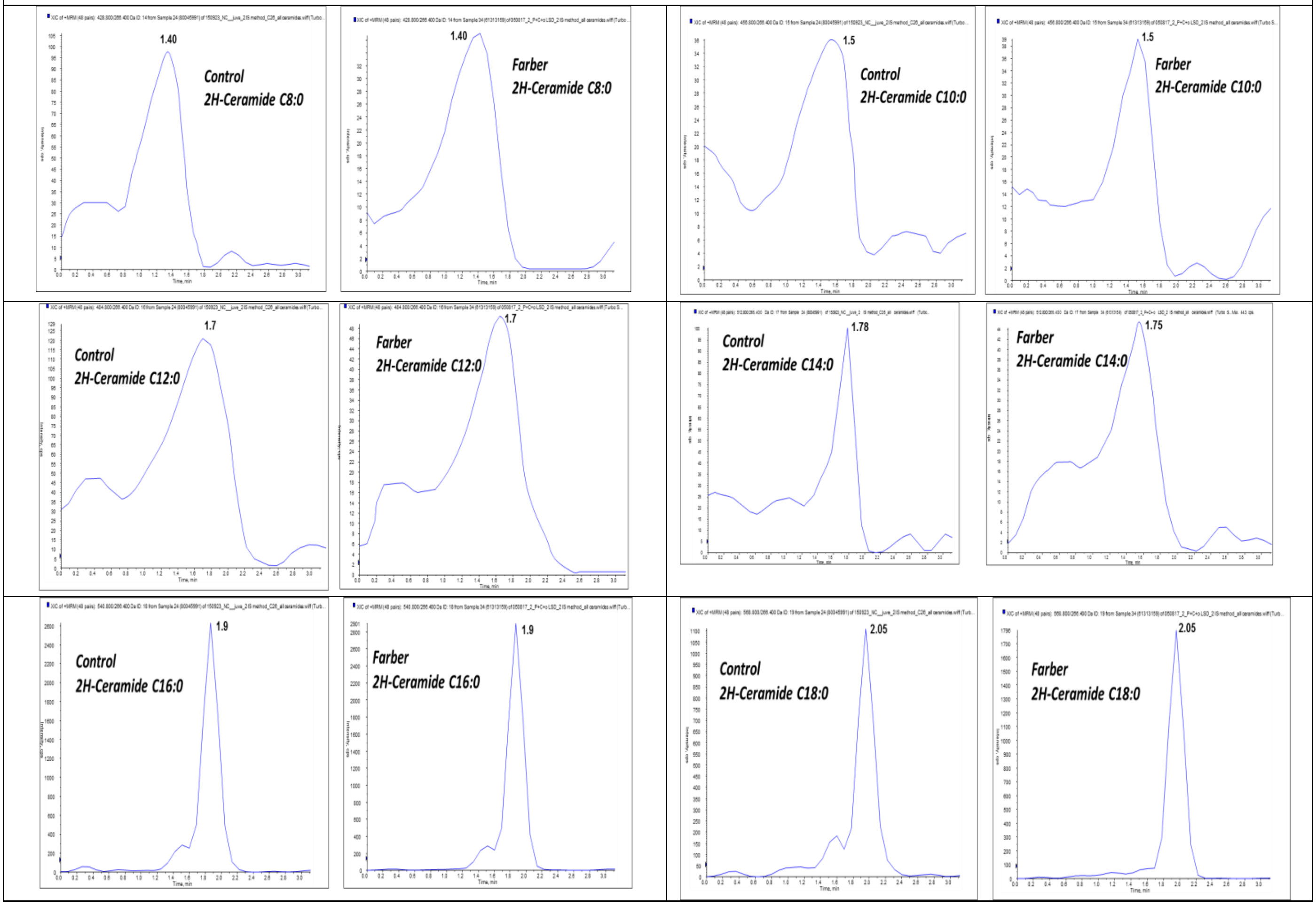

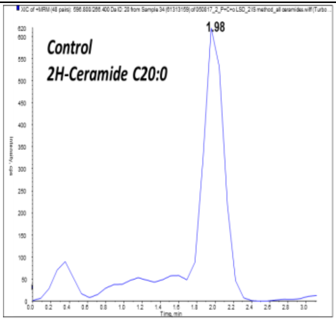

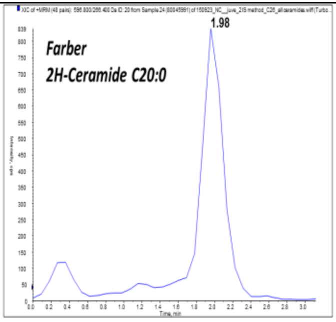

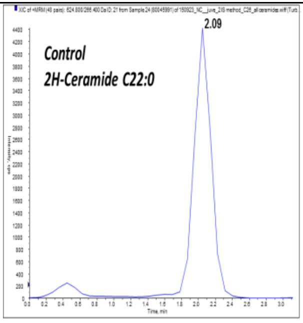

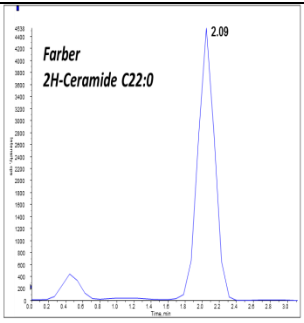

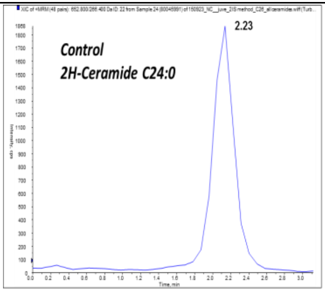

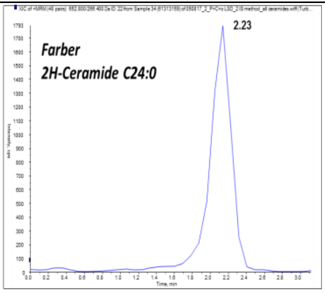

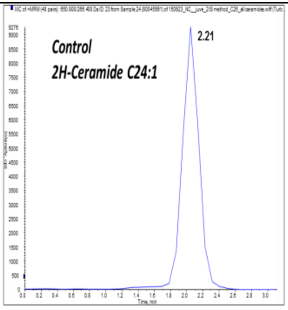

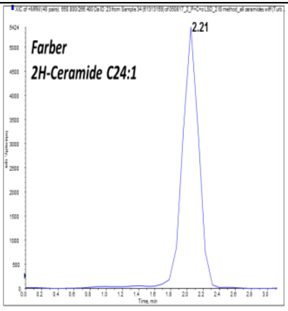

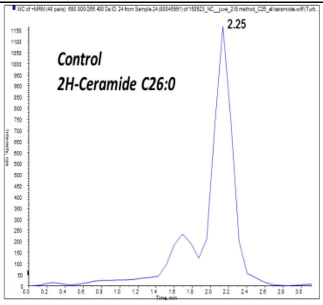

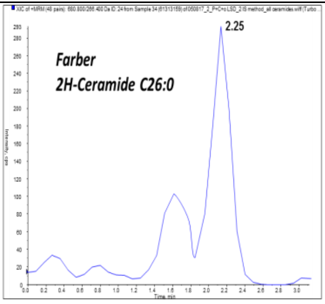

Glucosylceramides

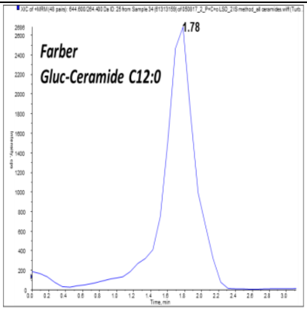

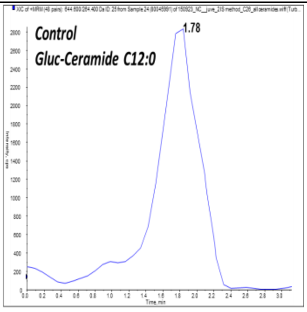

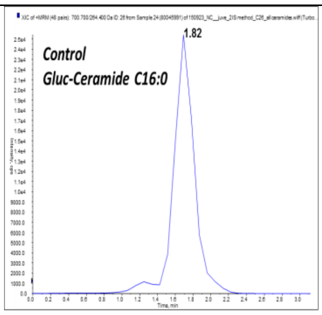

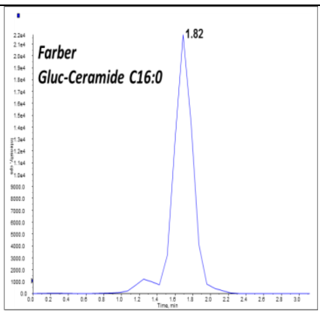

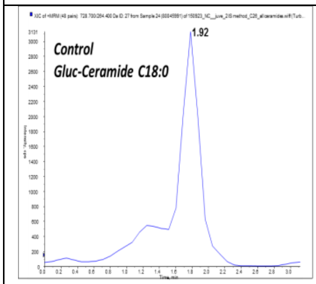

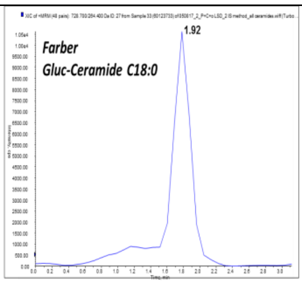

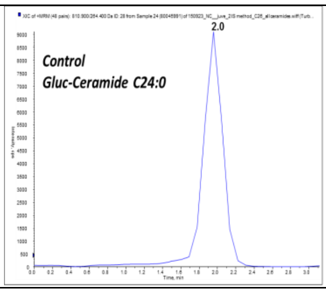

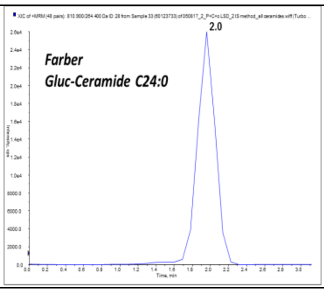

Dihydro-glucosylcermides

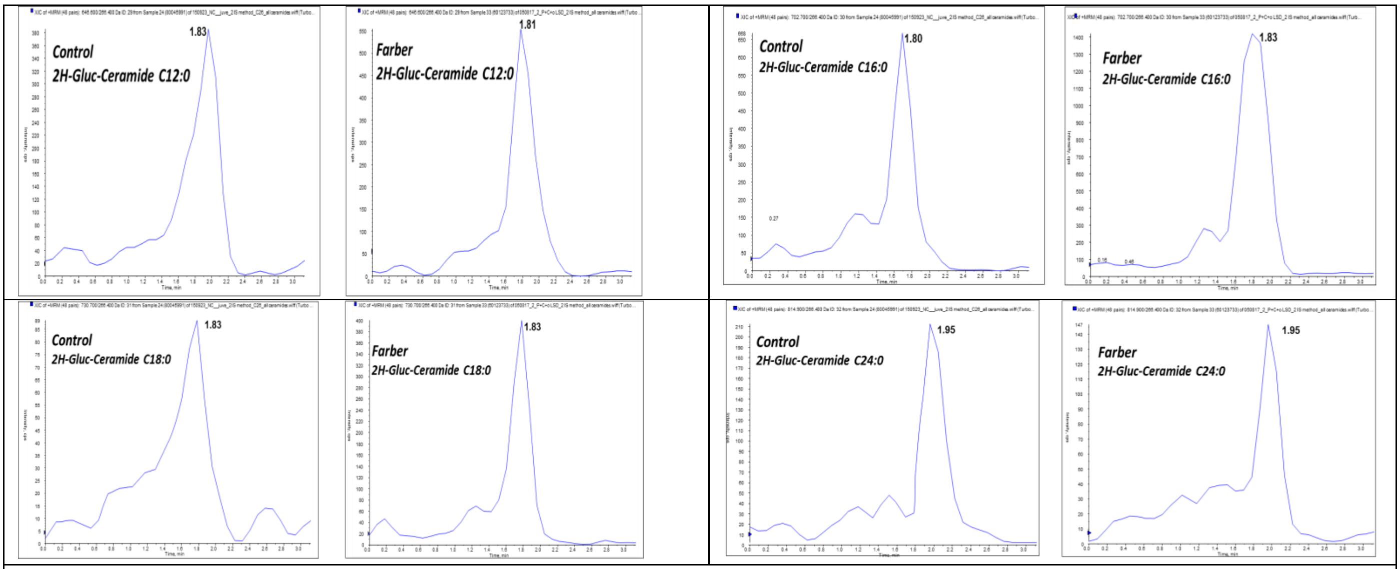

Sphingomyelins

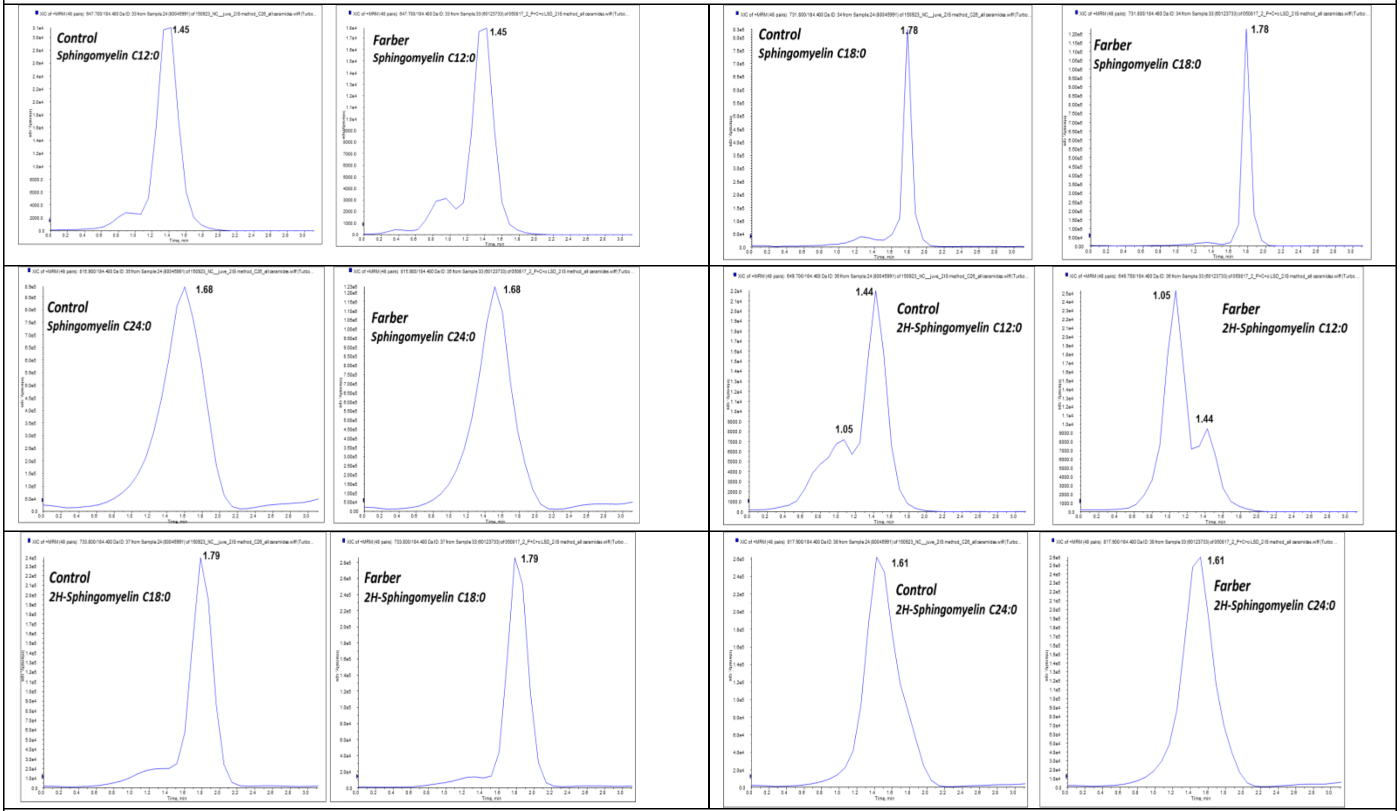

Other compounds

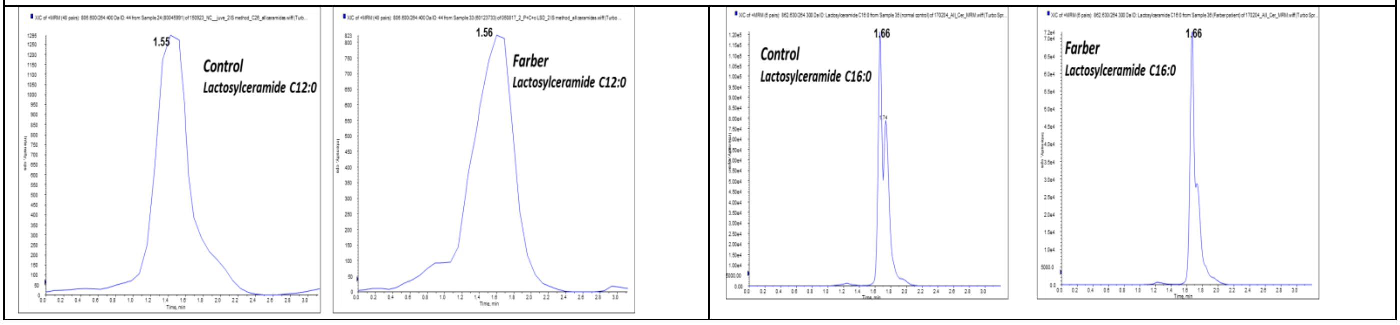

<table>
<tr>
<td>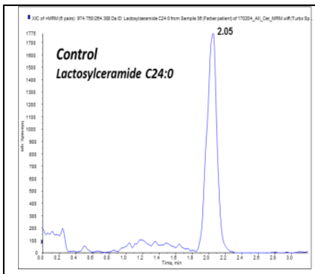

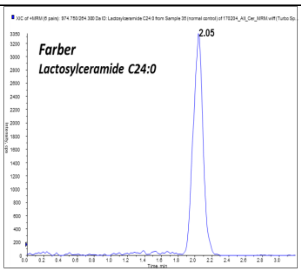
</td>
<td></td>
</tr>
</table>

Note regarding data processing for the samples found in very low concentrations (intensity of the signal under 500 counts: the TIC was smoothed to permit integration for the entire peak. For a better quantification of these analytes, the standard curve signals were smoothed with the same magnitude.

Supplementary Table S7. Quantification of ceramides and ceramides related molecules in DBS from healthy controls (NC), Farber Patients (FP), Farber carriers (FC) and patients with juvenile idiopathic arthritis (JIA)

The values are shown as mean ± standard deviation. *p-values (Mann-Whitney test) are only given for comparisons between FP vs. NC.

No.	Analyte	**NC (N=83)**	**FP (N=10)**	**FC (N=11)**	**JIA Patients (N=2)**	**p-value***
**Ceramides (µg/L blood)**						
A1.	Ceramide C4:0	4.3 ± 3.7	5.1 ± 0.9	3.5 ± 1.3	6.5 ± 1.3	0.1677
A2.	Ceramide C6:0	12.8 ± 2.8	9.5 ± 0.9	9.1 ± 1.5	6.9 ± 1.9	0.0436
A3.	Ceramide C8:0	40.9 ± 9.9	24.6 ± 2.4	22.9 ± 3.9	16.7 ± 8.8	0.0456
A4.	Ceramide C10:0	7.5 ± 1.4	7.3 ± 0.7	5.8 ± 1.8	3.6 ± 2.4	0.7948
A5.	Ceramide C12:0	4.8 ± 0.9	4.5 ± 0.7	3.5 ± 1.5	3.1 ± 0.9	0.9124
A6.	Ceramide C14:0	15.1 ± 3.3	14.9 ± 5.3	10.2 ± 2.1	13.9 ± 0.3	0.6852
A7.	Ceramide C16:0	222.1 ± 55.0	503.4 ± 209.1	334.3 ± 277.0	156.9 ± 222.0	0.6852
A8.	Ceramide C18:0	46.5 ± 10.7	89.5 ± 36.4	27.4 ± 25.1	57.9 ± 5.7	0.0398
A9.	Ceramide C20:0	23.9 ± 4.9	37.0 ± 11.1	27.2 ± 9.2	30.0 ± 7.2	0.0336
A10.	Ceramide C22:0	823.0 ± 239.4	541.2 ± 141.3	511.7 ± 109.0	405.1 ± 86.3	0.0417
A11.	Ceramide C24:0	2181.3 ± 441.9	1654.3 ± 365.9	1604.4 ± 837.1	601.3 ± 58.5	0.8705
A12.	Ceramide C24:1	702.5 ± 352.8	656.3 ± 134.5	668.6 ± 116.7	531.1 ± 145.1	0.8705
A13.	***Ceramide C26:0***	***33.1 ± 7.8***	***61.8 ± 21.8***	***53.2 ± 19.7***	***24.7 ± 4.4***	***< 0.0001***
**Total ceramides**		**4117.7 ± 1134.5**	**3609.3 ± 930.9**	**3311.8 ± 1406.2**	**1857.7 ± 544.9**	**0.4431**
**Dihydroceramides (µg/L blood)**						
A14.	Dihydroceramide C8:0	0.7 ± 0.5	0.5 ± 0.3	0.5 ± 0.2	0.3 ± 0.1	0.6317
A15.	Dihydroceramide C10:0	1.0 ± 0.4	0.5 ± 0.4	0.7 ± 0.3	0.7 ± 0.2	0.0509
A16.	Dihydroceramide C12:0	2.2 ± 0.5	1.6 ± 0.3	1.3 ± 0.2	1.6 ± 0.3	0.0514
A17.	Dihydroceramide C14:0	1.9 ± 0.5	1.5± 0.3	1.3 ± 0.3	1.8 ± 0.0	0.1020
A18.	Dihydroceramide C16:0	9.3 ± 2.1	17.8 ± 128.5	11.9 ± 10.4	13.3 ± 0.1	0.0302
A19.	Dihydroceramide C18:0	3.3 ± 1.3	9.3 ± 6.5	5.2 ± 2.9	4.3 ± 0.3	0.0451
A20.	Dihydroceramide C20:0	2.8 ± 0.7	3.5 ± 1.2	2.4 ± 1.0	2.0 ± 0.7	0.0761
A21.	Dihydroceramide C22:0	30.4 ± 7.5	23.0 ± 2.9	19.5 ± 4.5	16.0 ± 3.7	0.0830
A22.	Dihydroceramide C24:0	10.7 ± 5.5	13.0 ± 2.0	12.0 ± 2.6	9.4 ± 2.9	0.0714
A23.	Dihydroceramide C24:1	48.1 ± 12.2	43.1 ± 11.6	40.2 ± 18.7	16.1 ± 0.3	0.5026
A24.	Dihydroceramide C26:0	3.1 ± 0.7	2.5 ± 1.1	2.9 ± 1.4	2.6 ± 1.7	0.1640
**Total Dihydroceramide**		**113.5 ± 31.9**	**116.4 ± 38.1**	**97.9 ± 42.5**	**68.1 ± 10.3**	**0.5539**
**Glucosylceramide (µg /L blood)**						
A25.	Glucosylceramide C12:0	28.0 ± 5.8	42.2 ± 25.0	43.3 ± 35.9	19.8 ± 3.7	0.0528
A26.	Glucosylceramide C16:0	114.8 ± 30.4	206.9 ± 118.5	122.2 ± 106.3	91.5 ± 28.9	0.0569
A27.	Glucosylceramide C18:0	37.0 ± 13.4	46.9 ± 23.8	28.5 ± 12.7	18.0 ± 12.4	0.1594
A28.	Glucosylceramide C24:0	35.7 ± 26.1	94.9 ± 50.7	56.6 ± 47.7	46.1 ± 21.7	0.0397
A29.	Dihydroglucosylceramide C12:0	2.4 ± 0.7	3.2 ± 1.1	3.1 ± 1.1	1.1 ± 0.5	0.0535
A30.	Dihydroglucosylceramide C16:0	31.4 ± 7.2	20.4 ± 7.1	21.8 ± 5.9	7.3 ± 0.6	0.0644

A31.	Dihydroglucosylceramide C18:0	1.8 ± 0.5	2.1 ± 1 .1	1.6 ± 0.5	0.5 ± 0.3	0.5198
A32.	Dihydroglucosylceramide C24:0	1.3 ± 0.3	1.3 ± 0.5	1.4 ± 0.5	0.7 ± 0.2	0.3832
**Total Glucosylceramide**		**252.5 ± 84.5**	**417.9 ± 227.8**	**278.5 ± 210.5**	**185.0 ± 68.3**	**0.0520**
**Sphingomyelins (µg/L blood)**						
A33.	Sphingomyelin C12:0	358.0 ±111.1	319.1 ± 177.6	269.2 ± 116.3	309.5 ± 53.2	0.1285
A34.	Sphingomyelin C18:0	5302.4 ±1015.0	5300.7 ± 1138.9	4569.9 ± 772.7	5033.1 ± 141.2	0.7581
A35.	Sphingomyelin C24:0	34990.0 ± 13189.6	16933.2 ± 2429.1	19852.7 ± 1775.3	16629.6 ± 2340.5	0.0408
A36.	Dihydrosphingomyelin C12:0	613.5 ± 170.5	290.3 ± 32.7	250.8 ± 88.1	522.4 ± 54.9	0.0567
A37.	Dihydrosphingomyelin C18:0	3344.7 ± 992.1	2326.8 ± 432.2	2108.3 ± 467.6	2350.4 ± 36.3	0.0787
A38.	Dihydrosphingomyelin C24:0	6588.9 ± 1413.0	4335.8 ± 663.4	4508.3 ± 958.3	3637.7 ± 411.1	0.0058
**Total Sphingomyelins**		**51197.5 ± 16891.1**	**29505.8 ± 4873.9**	**31559.5 ± 4178.3**	**28482.7 ± 3037.3**	**0.0565**
**Other compounds (µg/L blood)**						
A39.	Lactosylceramide C12:0	6.2 ± 2.1	10.0 ± 4.1	7.3 ± 3.1	5.6 ± 0.6	0.0520
A40.	Lactosylceramide C16:0	1025.0 ± 264.8	942.1 ± 372.9	988.9 ± 248.5	834.1 ± 296.3	0.6046
A41.	Lactosylceramide C24:0	37.7 ± 19.7	42.0 ± 19.7	48.6 ± 26.8	61.1 ± 29.4	0.4798

Note: The values are shown as mean ± standard deviation. *p-values (Mann-Whitney test) are only given for comparisons between FP vs. NC.

Supplementary Figure S2. Normal distributions of C26 ceramide concentrations within normal cohort

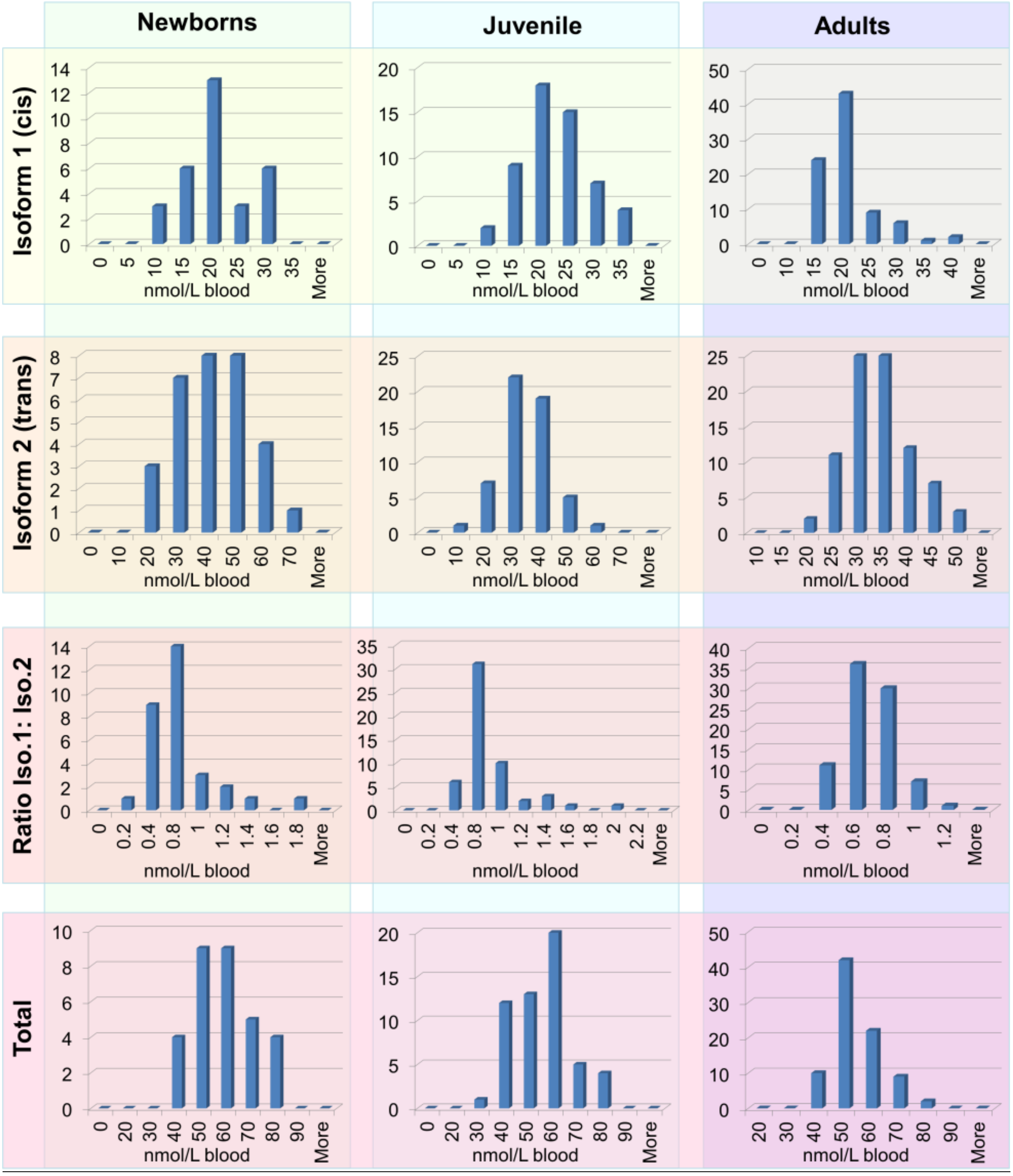

Supplementary Table S8. LC-MRM-MS parameters for the determination of C26 Ceramides

**LC parameters**								
Instrumentation	Waters UPLC Acquity							
Solvent A	50 mM formic acid							
Solvent B	50 mM formic acid in Acetone:Acetonitrile 1:1							
Column	ACE C8, 3µm, Ultra-Inert HPLC Column, 50x2.1mm (ACE,Germany)							
Flow	0.9 mL/min,							
	upstream to MS ; Waste to MS 2:1							
Gradient	Time (min)	0.0	0.2	1.7	1.8	2.8	2.9	3.1
	% B	60	60	100	100	100	40	40
Column temperature	60°C							
Equilibration time	0.1 minutes							
**MRM-MS parameters**								
Instrumentation	ABSciex 5500 TripleQuad							
Scans in period	118							
Relative start time	0.0							
Experiment in period	1							
Scan type	MRM							
Polarity	Positive							
Ion source	Turbo spray							
Resolution Q1	unit							
Resolution Q3	unit							
MR Pause	5.000 msec							
CAD	8 psi							
CUR	10 psi							
GS1	45 psi							
GS2	60 psi							
IS	5000 V							
TEM	200 °C							
EP	10 V							

**Transitions monitored**						
**ID**	***Q1 Mass (Da)***	***Q3 Mass (Da)***	***Dwell (msec)***	***DP(V)***	***CE(V)***	***CXP (V)***
**IS1**	624.3	282.2	100	30	38.4	11
**IS2**	664.5	264.4	200	34	46	13
**C26Cer**	678.7	264.4	200	34	46	13

Note: DP=declustering potential, CE=collision energy, CXP=collision cell exit potential.
